# The impact of dental care programs on healthcare system and societal outcomes: a scoping review

**DOI:** 10.1186/s12913-022-08951-x

**Published:** 2022-12-23

**Authors:** Abdulrahman Ghoneim, Arezoo Ebnahmady, Violet D’Souza, Kamini Kaura Parbhakar, Helen He, Madeline Gerbig, Sonica Singhal, Carlos Quiñonez

**Affiliations:** 1 Faculty of Dentistry, University of Toronto, Toronto, ON Canada; 2 Faculty of Dentistry, Dalhousie University, Halifax, NS Canada; 3 Faculty of Arts and Science, University of Toronto, Toronto, ON Canada; 4 Public Health Ontario, Toronto, ON Canada; 5 Schulich School of Medicine and Dentistry, London, ON Canada

**Keywords:** Dental care programs, Healthcare system, Societal outcomes

## Abstract

**Background:**

Dental diseases have detrimental effects on healthcare systems and societies at large. Providing access to dental care can arguably improve health outcomes, reduce healthcare utilization costs, and improve several societal outcomes.

**Objectives:**

Our objective was to review the literature to assess the impacts of dental care programs on healthcare and societal outcomes. Specifically, to identify the nature of such programs, including the type of services delivered, who was targeted, where services were delivered, and how access to dental care was enabled. Also, what kind of societal and healthcare outcomes have been attempted to be addressed through these programs were identified.

**Methods:**

We conducted a scoping review by searching four databases, MEDLINE, EMBASE, CINAHL, and Sociological Abstracts. Relevant articles published in English language from January 2000 to February 2022 were screened by four reviewers to determine eligibility for inclusion.

**Results:**

The search resulted in 29,468 original articles, of which 25 were included in the data synthesis. We found minimal evidence that answers our proposed research question. The majority of identified programs have demonstrated effectiveness in reducing medical and dental healthcare utilization (especially for non-preventive services) and avert more invasive treatments, and to a lesser degree, resulting in cost-savings. Moreover, some promising but limited evidence about program impacts on societal outcomes such as reducing homelessness and improving employability was reported.

**Conclusion:**

Despite the well-known societal and economic consequences of dental problem, there is a paucity of studies that address the impacts of dental care programs from the societal and healthcare system perspectives.

**MeSH terms:**

Delivery of Health Care, Dental Care, Outcome assessment, Patient acceptance of Health Care.

**Supplementary Information:**

The online version contains supplementary material available at 10.1186/s12913-022-08951-x.

## Introduction

Oral diseases are one of the most common chronic conditions affecting individuals, which can be extremely painful and debilitating, causing significant morbidity in a number of cases [1–3]. In addition to the detrimental effects it has on individuals, the broader impacts it bears on the healthcare system and society at large have also been documented in the literature. The most commonly cited consequences of unaddressed dental problems include visiting hospital emergency rooms for non-traumatic dental problems [4–7], loss of productivity [8], as well as worsened academic and employment performances [9–12]. Analysis from the United States 1981 National Health Interview survey results conducted by Gift et al. [13] and Reisine [14] have shown that dental conditions resulted in around 17 million days of restricted activity, 8 million days of bed disability, and 7 million days of work loss.

The impacts of dental problems are far more significant in the current times. According to the latest statistics, around 92 million work or school hours across the United States are lost annually due to unplanned (emergency) dental care [15]. Similarly, in Canada, 40 million hours are lost annually by working individuals due to dental problems and treatments, in addition to 20 million school hours also lost by children citing the same reason [8]. From the healthcare perspective, millions of Canadians who lack access to dental services visit physicians’ offices or hospitals’ emergency departments for non-traumatic dental problems, every year [16]. Studies have also shown that children with worse oral health are more likely to miss school days and have poorer academic performances compared to their counterparts who do not experience dental problems. This results in a collective financial impact of more than 1 billion dollars annually [8].

Globally, the annual indirect costs (i.e., loss of productivity) due to major dental diseases (tooth decay, periodontal diseases, and severe tooth loss) amounted to over 144 billion dollars in 2010. The authors argue that these significant economic losses rank dental diseases as one of top 10 deadliest diseases [17]. To note, these costs do not include more severe and costly conditions like cancer dysplasias of the oral mucosa, oral infections, oral developmental disorders (e.g., clefts of the lip and palate), and noma.

Owing to the highly privatized dental care systems in countries such as Australia, Canada, Italy, and the United States [18], income-related inequalities persist and access to necessary dental services remains a challenge for many, resulting in a significantly disproportionate burden for the vulnerable and marginalized communities [19–24]. These inequalities in oral health and barriers to access to dental have been further underscored during the COVID-19 pandemic, which has instigated national, and international policy discussions to explore and consider broader population coverage for dental care [25–29]. Nonetheless, much work remains to be done.

Providing dental care through various dental care programs may be one way to avert such costly impacts. However, despite our knowledge of how detrimental poor oral health is, we lack the empirical evidence that demonstrates how improving access to dental care can influence healthcare and societal outcomes. Therefore, this study aims to map the evidence on how providing dental care through various dental programs can influence the healthcare system and societal outcomes. The objective of this review is to address the following PICO question:

What are dental care programs’ healthcare and societal impacts on their beneficiaries?

Specifically, to highlight the following:

1. Identify the types of dental programs and types of healthcare and societal outcomes that are addressed through such programs
2. What is the mechanism of access adopted by the programs identified?
3. How successful were these programs in improving the associated outcomes?

## Methods

### Design and study search

To address these questions and in collaboration with the librarians at the University of Toronto, we systematically searched four electronic databases, Ovid (MEDLINE), EMBASE, CINAHL, and Sociological Abstracts using the search strategy outlined in Additional file 1. We also reviewed the reference lists of eligible articles to include any articles that might have been missed during the original search. In addition, we reviewed grey literature using Google Scholar, as well as online repositories of international, national, and provincial dental organizations, and organizational guidelines, reports, position statements, clinical trial registries, and websites. The search was restricted to English language articles published from January 2000 to February 2022. Given the fast-evolving nature of evidence, we decided to exclude studies conducted before the turn of the millennium as results would likely be outdated and irrelevant to today’s context.

### Inclusion and screening process

We included articles investigating the impact of dental care programs on any age group from qualitative, quantitative, or mixed methods research studies. For quantitative research, the following study designs were included: controlled/uncontrolled trials, observational including cohort studies (prospective or retrospective), and systematic reviews and meta-analyses. We included articles that had an outcome relevant to the healthcare or societal levels.

We included only those dental care projects/programs that provided dental benefits, with the aim to improve oral health outcomes, and/or reduce burden of illness, injury, or disability. Dental care programs that were delivered directly (clinical care) to participants, including preventive, restorative, both preventive and restorative met the inclusion criteria. Thus, we excluded studies that investigated the impact of population level interventions—defined as policies, programs and resource distribution approaches that impact a number of people by changing the underlying conditions of risk and reducing health inequities [30]—but had no direct clinical dental care intervention (e.g., community water or salt fluoridation, oral health education alone). Studies with insufficient data (e.g., in conference abstract or books) and the cross-sectional design (exposure and outcome are simultaneously assessed) were also excluded.

### Selection and data extraction

After removing the duplicates, all unique articles were moved to Covidence systematic review software [31] and given an identification code. First, four calibrated reviewers (AE, AG, VD, KKP) independently screened titles, abstracts, and keywords for relevance. The agreement among these four reviewers was substantial (Fleiss Kappa = 0.7) [32]. Relevant records proceeded to the second step, which involved screening the full text of the relevant articles. In both steps, articles were reviewed against the inclusion/exclusion criteria. To avoid redundancy, when more than one publication from the same study were identified, the team selected the most recent one only. All discrepancies were resolved via discussion until consensus was reached.

All potential selected articles proceeded for quality appraisal. Two independent reviewers (AE and VD) assessed the quality and relevance of each article to the study question. Unlike systematic reviews, quality assessment in a realist and scoping reviews is not based on the methodological design hierarchy [33]. Therefore, for our review the quality and scoring of the potentially eligible articles were assessed using a pre-designed quality assessment form (for qualitative, quantitative and mixed methods studies) from Minian et al. [34]. The overall quality score of each study was assessed by using the quality assessment tool outlined in Additional file 2. Studies were evaluated based on a series of 8–10 questions, depending on their respective design. Based on the assessment of those criteria, descriptors of 1 star (0–25%), 2 stars (26–50%), 3 stars (51–75%), and 4 stars (76% +) were assigned. To maintain the highest level of quality, only those studies scoring 4 stars were included. Again, any discrepancies were resolved through discussion until consensus was achieved.

## Results

**Fig. 1:**
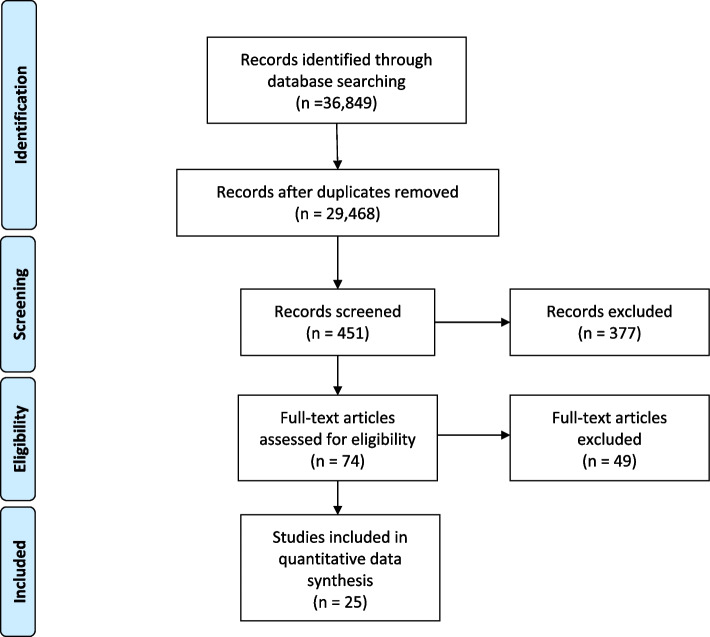
PRISMA flow diagram

We identified 29,468 records, screened 451 papers for the full-text review, and 74 studies met the inclusion criteria. Of them, 49 studies were removed as they did not fit our inclusion criteria after full-text review and for having a quality score was below four stars making the final number of selected studies at 25. Insufficient description of sample size and/or randomization process (if applicable), apparent biases, and use of non-validated measures were the prominent reasons for studies to receive less than four stars. Figure 1 depicts the PRISMA flow diagram of the included articles.

### Characteristics of the included articles

The articles included in this review were classified according to the date of publication, the jurisdiction in which the program was implemented, the targeted population, the type of dental services provided, the setting in which it was provided, and finally the mechanism of access to dental care, based on the Penchansky and Thomas’ theory of access [35].

We also summarized the studies based on the impacts the programs have on the outcomes of interest (healthcare system and societal outcomes). Table 1 outlines the characteristics of the included studies. The majority of studies were published after the year 2010, in the United States, targeted children, and examined the healthcare systems impacts. Only three studies probed the societal impacts of dental care programs. The most common type of program implemented was preventive and implemented in a conventional dental setting. Other common settings included community based and medical/hospital settings. Table 2 provides additional details about the characteristics of included studies.

**Table 1:** Characteristics of included studies

	n (%)
**Year of publication**	
2000–2010	4 (16)
2011–2022	21 (84)
**Country/jurisdiction**	
United States	17 (68)
Finland	2 (8)
Canada	1 (4)
Sweden	1 (4)
South Africa	1 (4)
Scotland	1 (4)
Systematic reviews	2 (8)
Targeted population*	
Children	13 (48)
Adults	11 (41)
Elders	2 (7)
All age groups	1 (4)
**Outcome level**	
Healthcare system outcome	22 (88)
Societal outcome	3 (12)
Study setting*	
School-based setting	4 (14)
Community-based setting	5 (18)
Medical/hospital settings	3 (11)
Dental settings	11 (39)
Any setting	1 (4)
Not specified	4 (14)
**Types of services provided**	
Diagnostic and preventive services	10 (40)
Primary dental care	1 (4)
Referral services	2 (8)
Comprehensive care/any dental care	3 (12)
Non-emergency and routine dental care	2 (8)
Not specified	7 (28)
Mechanism of access*	
Affordability	17 (57)
Accessibility	1 (3)
Availability	2 (7)
Acceptability	3 (10)
Accommodation	1 (3)
Not specified/Not applicable	6 (20)

^*^ Total percentages might add to more than 100% as they were addressed in more than one study

**Table 2:** Detailed characteristics of included studies

Author(s), year	Country/Jurisdiction	Targeted population	Type of services	Setting	Mechanism of access
1. Abdus et al., 2019 [36]	United States	Medicaid adults	Not specified	Dental setting	Affordability
2. Beil et al., 2012 [37]	United States	Medicaid children	Preventive services	Dental setting Community setting Hospital setting	Affordability
3. Bergström et al., 2016 [38]	Sweden	Children (12–15 years old)	Preventive services	School setting	Not specified
4. Bhayat et al., 2016 [39]	South Africa	Everyone	Primary dental care	Dental setting Medical setting	Affordability
5. DiMarco et al., 2010 [40]	United States	Homeless children	Referral services	Community setting	Affordability Availability Accessibility Acceptability
6. Elani et al., 2020 [41]	United States	Medicaid adults	Not specified	Not specified	Affordability
7. Elani et al., 2020 [42]	United States	Medicaid adults	Not specified	Not specified	Affordability
8. Kaakko et al., 2002 [43]	United States	Children (1–4 years old)	Preventive services	Dental setting	Affordability
9. Khouja et al., 2020 [44]	United States	Medicaid low-income parents	Not specified	Not specified	Affordability
10. Kidd et al., 2020 [45]	Scotland	Children	Diagnostic and preventive services	School and community settings	Affordability
11. Lyu et al., 2020 [46]	United States	Medicaid low-income adults	Non-emergency dental services (not specified)	Not specified	Affordability
12. Maserejian et al., 2008 [47]	United States	Children (6–10 years old)	Preventive services	Community setting	Affordability
13. McQuade et al., 2011 [48]	United States	Medicaid children (6 years and younger)	Preventive services	Dental setting	Not specified
14. Metsch et al., 2015 [49]	United States	Low-income adults with HIV	Referral services	Dental setting	Acceptability
15. Moeller et al., 2020 [50]	United States	Medicare Adults and elders	Routine dental care (not specified)	Not specified	Affordability
16. Nihtilä et al., 2013 [51]	Finland	Adults	Not specified	Dental setting	Affordability
17. Nowak et al., 2014 [52]	United States	Children (7 years and younger)	Not specified	Dental setting	Not specified
18. Nunez et al., 2013 [53]	United States	Homeless Veterans (adults and elders)	Comprehensive dental care	Community setting	Not specified
19. Pourat et al., 2020 [54]	United States	School children	Preventive services and referrals	School setting	Affordability
20. Rozier et al., 2010 [55]	United States	Low-income children	Preventive services	Medical setting	Acceptability Accommodation Availability
21. Sanjeevan et al., 2019 [56]	Systematic review	Children (under the age of 15 years old)	Diagnostic services	School setting	Not specified
22. Sen et al., 2013 [57]	United States	Children	Preventive services	Dental setting	Affordability
23. Singhal et al., 2013 [11]	Systematic review	Adults with problems accessing dental care	Any dental care	Any setting	Not specified
24. Singhal et al., 2016 [58]	Canada	Adults enrolled in social assistance programs	Any dental care	Dental setting	Affordability
25. Suominen et al., 2000 [59]	Finland	Young adults	Not specified	Dental setting	Affordability

### Targeted population and implementation settings

The identified studies exhibited a wide variation in the populations benefiting from the programs; however, children were targeted the most (*n* = 13, 52%) [37, 38, 40, 43–45, 47, 48, 52, 54–57], particularly those children, who were from economically and socially disadvantaged background. For example, five studies (20%) targeted children from low-income families (mostly Medicaid eligible) [37, 43, 44, 48, 52], one study targeted children living in rural areas [47], and one study examined the impact on homeless children [40]. The second most common age group targeted were adults (*n* = 11); particularly, Medicaid adults [36, 41, 42, 44, 46, 50], adults living with human immunodeficiency virus infection [49], and adults enrolled in social assistance programs [58]. One study assessed the impacts of providing dental care on homeless adults and elder veterans [53].

In terms of settings, four programs were implemented in a school setting [38, 45, 54, 56] and found that providing diagnostic (dental screening) [56], and preventive services (fluoride varnish and referrals) [38, 45, 54] were effective in averting a significant number of future restorative treatments [38, 54] and increase dental care utilization [38, 45, 56].

Seven studies from the United States examined the impacts of existing or expanded Medicare and Medicaid programs on the utilization of healthcare and emergency department due to dental problems [36, 37, 41, 42, 44, 46, 50]. In the current context of healthcare in the United States, dental care is predominately provided in privately owned dental clinics and privately funded services on a fee for service basis. Under the Affordable Care Act (ACA), many of the state programs either created or expanded their dental programs allowing their eligible beneficiaries to receive publicly financed dental care services.

Other common settings where programs where delivered were in community health centers (*n* = 5, 20%) [37, 40, 45, 47, 53], and hospital/medical settings (*n* = 3,16%) [37, 39, 55]. For instance, Rozier and colleagues [55] examined the impacts of a North Carolina program that reimbursed physicians for providing preventive oral health services for children during their regular physical exams in first three years of life. Maserejian et al. [47] and Nunez et al. [53] assessed programs delivered in shelters and homeless community centers.

### Types of services provided

As Benjamin Franklin once said, “*one ounce of prevention is worth a pound of cure*,” many of today’s dental programs still adopt the same approach when addressing oral health issues. The majority (*n* = 10, 40%) [37, 38, 43, 45, 47, 48, 54–57] of identified studies implemented a preventive program which included an array of services such a fluoride varnish, scaling, polishing, and dental sealants. Three studies (12%) considered comprehensive dental program or any type of dental care including services like restorations, extractions, and prosthodontic care [11, 53, 58], and two programs provided non-emergency and routine dental care [46, 50]. Seven studies (28%) did not specify the type of services provided [36, 41, 42, 44, 51, 52, 59].

### Program outcomes and effectiveness

We identified three healthcare system and two societal level outcomes (Additional file 3). Healthcare system outcomes captured were 1) the utilization of dental and/or healthcare services, 2) healthcare expenditure and cost savings, and 3) the number and type of averted treatments/services. The societal level outcomes captured were changes in homelessness status [53] and employability [11, 58]. Main findings extracted from the included studies are outlined in Table 3. In terms of dental and healthcare services utilization, eleven studies demonstrated positive impacts [36, 39, 40, 42, 45, 46, 48, 51, 54–56]. The results ranged from a 5% increase in preventive dental visits and use of major dental treatments (Lyu et al. 2020) [46] to 43% of families securing dental appointments after receiving shelter-based care (DiMarco et al. 2010) [40], and 46% increase in dental appointments after the introduction of primary dental health services (Bhayat et al. 2003) [39].

**Table 3:** Main findings from included studies

Author(s), date	Main study findings
1. Abdus et al., 2019 [36]	Compared with Medicaid enrollees in states that did not provide coverage, enrollees in states that provided coverage of nonemergency dental services were approximately 9 percentage points more likely to have a dental visit, approximately 7 percentage points more likely to have any preventive dental service, and more likely to have all other types of dental services except oral surgery services. The out-of-pocket share of dental expenditure, among Medicaid enrollees with visits, was approximately 19 percentage points lower in covered states than in uncovered states. This difference was equal to approximately one-half of the out-of-pocket share of dental expenditures in uncovered states (38.50%).
2. Beil et al., 2012 [37]	Children who had a primary or secondary preventive visit by age 18-months had no difference in subsequent dental outcomes compared to children in older age categories. Among children with existing disease, those who had a tertiary preventive visit by age 18-months had lower rates of subsequent treatment (18–24 months IDR: 1.19, [95% CI: 1.03–1.38]; 25–30 months IDR: 1.21, [95% CI: 1.06 – 1.39]; 37–42 months IDR: 1.39, [95% CI: 1.22 – 1.59]) and lower treatment expenditures compared to children in older age categories. Children with existing disease who received a tertiary preventive visit by age 18 months had 19% to 39% fewer treatments per time enrolled and were predicted to have $38-$138 fewer treatment related expenditures per year from age 3½ to 6 years than children who had tertiary preventive visits at older ages.
3. Bergström et al., 2016 [38]	Caries prevalence and caries increment in 15-year-olds were significantly lower after the implementation of the programme. Group 2, without a programme, had the highest caries increment. This means that it is possible that the fluoride varnish programme, during this four-year period, prevented fillings for a total cost of 391 SEK for each individual taking part.
4. Bhayat et al., 2016 [39]	There was a mean 46% increase in attendance after primary dental health services were introduced, with more than a sixfold increase in casual attendees (pain, sepsis) than in booked patients (restorative treatment, dentures, orthodontics).
5. DiMarco et al., 2010 [40]	Shelter-based care was effective in improving access: 43% of families secured dental appointments and perceived access barriers decreased after shelter-based care (t = 54.695; p ≤ 0.001).
6. Elani et al., 2020 [41]	The Affordable Care Act (ACA) increased rates of dental coverage by 18.9 percentage points in states that provide dental benefits through Medicaid. In terms of utilization, expansion states that provide dental benefits saw the greatest increase in people having a dental visit in the past year (7.2 percentage points). However, there was no significant change in the overall share of people who had a dental visit in the past year, although the expansion was associated with a significant increase in this metric among White adults.
7. Elani et al., 2020 [42]	In states that expanded Medicaid and offered dental coverage, dental Emergency Department (ED) visits decreased by 14.1 percent (from 19,443 to 16,709, for a net difference of 2,734). By contrast, in the remaining three state groups, dental ED visits rose. Meanwhile, the expansion significantly increased Medicaid coverage and decreased the rate of self-pay for ED dental visits.
8. Kaakko et al., 2002 [43]	Utilization is described in two periods: the first period was February l, 1997, to January 31,1998, and the second period was February 1, 1998, to May 31, 1999. In the first period the utilization rate (based on one or more dental claims) was significantly higher for the ABCD group than for the group of Medicaid-enrolled children not in ABCD (34.0% vs 24.7%; chi-square = 4.5; *P* = .03) (Table 1). During the second period, there was no statistically significant difference in utilization rates between ABCD and Medicaid-enrolled children not in ABCD. There were no statistically significant differences in overall expenditures for dental care between the groups in either period. During the first period, annual dental care expenditures were $67.32 for ABCD children and $52.44 (P = .35) for Medicaid enrolled children not in ABCD, respectively.
9. Khouja et al., 2020 [44]	Over the study period, 37.8 percent of low-income children received at least one annual preventive dental visit. We found no change in children's receipt of preventive dental care associated with Medicaid expansions in states that covered (1.26 percentage points; 95% CI: − 3.74 to 6.27) vs did not cover preventive dental services for adults (3.03 percentage points; 95% CI: − 2.76 to 8.81). (Differential change: − 1.76 percentage points; 95% CI: − 8.09, 4.56). However, our estimates are imprecise, with wide confidential intervals that are unable to rule out sizable effects in either direction. We did not find an association between Medicaid expansions with concurrent coverage of preventive dental services for adults and children's use of these services. Factors other than parental access to dental benefits through Medicaid may be more salient determinants of preventive dental care use among low-income children.
10. Kidd et al., 2020 [45]	The universal interventions had high population reach: nursery toothbrushing (89.1%), dental practice visits (70.5%). The targeted interventions strongly favored children from the most deprived areas: Dental Health Support Worker (DHSW) contacts (Scottish Index of Multiple Deprivation (SIMD) 1: 29.5% vs SIMD 5: 7.7%), nursery Fluoride Varnish Applications (FVAs) (SIMD 1: 75.2% vs SIMD 5: 23.2%).
11. Lyu et al., 2020 [46]	Expanding Medicaid in 2014 with extensive or limited dental coverage increased preventive dental visits and use of major dental treatments by over 5 percentage-points in 2014 and 2015. The increase in preventive visits continued in 2016 in expanding states with extensive coverage, while increase in major dental treatments continued in 2016 in expanding states with limited coverage. There is some but less consistent evidence of an increase in dental treatment with emergency-only coverage
12. Maserejian et al., 2008 [47]	On average, urban children utilized 69 percent of the visits and rural children utilized 82 percent of the visits. For both sites, utilization steadily decreased until the end of the 5-year trial. Among these children with unmet dental needs, the provision of free preventive dental care was insufficient to remove the disparities in utilization and did not consistently result in high utilization through follow-up.
13. McQuade et al., 2011 [48]	While the RIte Smiles (Rhode Island’s managed oral health program) began enrolling children in September 2006, several initiatives were underway beginning in 2004 that could have impacted utilization of dental care. As such, there appears to have been a slight trend upward on dental care between 2002 and 2004; however, the major inflection points in both participation and utilization appear between 2005 and 2007—coinciding with implementation of the RIte Smiles program. In fact, there was a 28% increase in overall participation in dental care between 2005 and 2010, a 33% increase in preventive visits and a 50% increase in treatment visits.
14. Metsch et al., 2015 [49]	The odds of having a dental care visit were about twice as high in the intervention group as in the standard care group at 6 months (adjusted odds ratio [OR] = 2.52; 95% confidence interval [CI] = 1.58, 4.08) and 12 months (adjusted OR = 1.98; 95% CI = 1.17, 3.35), but the odds were comparable in the 2 groups by 18 months (adjusted OR = 1.07; 95%CI = 0.62, 1.86). We demonstrated that a dental case management intervention targeting people with HIV was efficacious but not sustainable over time.
15. Moeller et al., 2020 [50]	Our preliminary 2-year time frame investigation does not provide evidence that a Medicare dental benefit covering routine care would have cost savings by lowering medical care use and expense of the elderly. We instead found that annual use of preventive dental care by older dentate persons is correlated with higher annual use and expense for office-based visits and, as a result, with higher overall health care utilization and expenditures. We also found that older persons currently using routine dental care have healthier lifestyles and greater access to care and use of preventive medical care than current nonusers.
16. Nihtilä et al., 2013 [51]	Most heavy users (61.6%) became low users and only 11.2% remained chronic heavy users. Most low users (91.0%) remained low users. For heavy users, the mean number of dental visits per year (3.0) during the follow-upperiod was significantly lower than initially in 2004 (8.3) (*p* < 0.001) but 74.8% of heavy users had had emergency visits compared with 21.6% of the low users (*p* < 0.001). A third (33%) of the visitors in each group had no proper examination and treatment planning during the 5-year follow-up period and two or more examinations were provided to fewer than half of the heavy (46.1%) or low (46.5%) users.
17. Nowak et al., 2014 [52]	Of 42,532 subjects, 17,040 (40 percent) were early starters and 25,492 (60 percent) were late starters. There were 3.58 more dental procedures performed on late starters, over eight years of follow-up, than on early starters (*P* < .001). Late starters spent $360 more over eight years of follow-up than early starters (*P* < .001).
18. Nunez et al., 2013 [53]	Veterans who received dental care were 30% more likely than those who did not to complete the program, 14% more likely to be employed or financially stable, and 15% more likely to have obtained residential housing. Provision of dental care has a substantial positive impact on outcomes among homeless veterans participating in housing intervention programs. This suggests that homeless programs need to weigh the benefits and cost of dental care in program planning and implementation.
19. Pourat et al., 2020 [54]	We found a reduction in ratio of treatment (particularly restorative) to total services in the fourth year, given receipt of portable preventive care in the third year (direct impact) and receipt of portable preventive care in prior years (indirect impact). Older children and those covered by Medicaid (versus privately insured) had a higher ratio of treatment to total services in the fourth year. Our retrospective analysis showed CHC portable dental program may reduce the use of treatment services over time among underserved children.
20. Rozier et al., 2010 [55]	The data set included more than eleven million child-month records (in other words, one record for each month) for 629,005 Medicaid-enrolled children ages six months up to three years during the period 2000–2006. Data in Exhibits 1 and 2 reflect the gradual implementation of the program over the seven-year study period as more physicians and staff received the required training. The number of both well-child visits and oral health visits in medical offices per hundred Medicaid enrolled children increased over time within every age group (Exhibit 1). The increase was largest for children ages 12–23 months (66.2 oral health medical visits per hundred Medicaid-enrolled children). Two additional measures (not shown) reflect differences in how many children were seen by the program by age group. The percentage of children with at least one oral health medical visit in 2006 was 19.4 percent for ages 611 months, 38.8 percent for ages 12–23 months, and 17.8 percent for ages 24–35 months. The percentage of well-child visits that included oral health services in 2006 was 16.2 percent for ages 6–11 months, 35.8 percent for ages 12–23 months, and 43.0 percent for ages 2435 months, which suggests a greater likelihood of oral health services for two-year-old children if they had a well-child visit. Analysis of physician and dentist Medicaid claims from period 2000–2006 shows that the program greatly increased preventive oral health services. By 2006 approximately 30 percent of well-child visits for children ages six months up to three years included these services.
21. Sanjeevan et al., 2019 [56]	Five studies met the inclusion criteria, covering a population of 28,208 school children of which 21,447 were included in the meta-analysis. The review concludes that school based dental screening marginally increases the dental attendance by 16 percent as opposed to a non-screening group (RR 1.16 (95% CI 1.11, 1.21). The quality of evidence was found to be low.
22. Sen et al., 2013 [57]	Using data on Children’s Health Insurance Program (CHIP) enrollees in Alabama, we found that preventive dental visits reduce a child’s subsequent non preventive dental visits and expenditures compared with years when the same child had no preventive visits. Restorative services obtained during preventive visits further reduced subsequent non preventive dental visits and expenditures. However, we found no evidence that preventive dental visits generate net savings for the program, at least in the 2-year follow-up period of our study.
23. Singhal et al., 2013 [11]	Seven articles were considered eligible for this review. They varied in study design, target population and intervention studied. Overall, they presented low levels of evidence due to small sample sizes, lack of control groups, combined interventions or being based on anecdotal reports. There is a limited amount of evidence concerning the assumption that dental care can improve employment outcomes. The scarcity of well-conducted studies and the poor quality of evidence makes it difficult to judge the effect of dental care on employment outcomes. More studies need to be conducted in order to confirm or dismiss this generalized assumption.
24. Singhal et al., 2016 [58]	We received data for 8,742 people (2,742 treatment, 6,000 no-treatment). At one year, employment outcomes were not significantly different between the two groups (adjusted odds ratio = 0.93; 95% CI: 0.83–1.03). Post-hoc analysis shows that the change in proportion of individuals leaving social assistance for employment over time was significantly higher (*p* = 0.0014) among those receiving treatment (13–29%; 124% increase) than those not receiving treatment (18–33%; 83% increase).
25. Suominen et al., 2000 [59]	While the total number of young adults who had received reimbursement for private dental care increased from about 53,000 (1986) to 200,000 (1994) due to extended eligibility, the number of users in the youngest group decreased from 53,000 to 23,000. Attending infrequently (1–2 times during the study period) was most common among the youngest adults and frequent attendance (annually) was most common among older adults. The annual mean cost was slightly lower among the frequent attenders in almost every cohort. Variation in the mean number of annual visits was directly correlated with costs. Frequent attenders most often received diagnostic and preventive measures while restorations and surgery were most common for the infrequent attenders.

The evidence for cost savings and reduced expenditure was overall weaker and inconclusive, as only six studies examined these outcomes [36–38, 50, 52, 57]. Abdus et al. [36] reported a 19% reduction in the out-of-pocket dental expenses in Medicaid enrollees in covered states compared to those in uncovered states and Nowak et al. [52] showed that early starters of dental visits incurred $360 less dental expenses over the 8 years of follow-up compared to late starters. Another example of programs that demonstrated cost savings was the school-based fluoride varnish program implemented for adolescents in Sweden. Bergström et al. stated that during four-year period, the program prevented fillings for a total cost of 391 Swedish Krona (SEK) for each individual taking part [38]. On the contrary, Sen et al. reported that more preventive visits did not reduce overall dental, or medical (inclusive of dental) expenditures [57]. In addition, Moeller and colleagues showed that increased dental visits has, in fact, resulted in increased medical expenses, albeit that being for preventive medical procedures, in their 2-year follow-up analysis frame [50].

Finally, seven studies examined the impacts of implementing dental programs on averting future treatments. For example, Beil et al. stated that children with existing disease who received a tertiary preventive visit by age 18 months had 19% to 39% fewer treatments per time enrolled from age 3½ to 6 years than children who had tertiary preventive visits at older ages [37]. Similarly, Nowak et al. reported an average of 3.58 more dental procedures performed on late starters of the dental program, compared to early starters, over the eight years of follow-up [52]. Findings from other studies by Beil et al. [37], Lyu et al. [46], Sen et al. [57], Pourat et al. [54], and Suominen et al. [59], also corroborate those findings and emphasize the importance of investing in dental programs to prevent future, more extensive and costlier treatments.

### Mechanisms of access

According to Penchansky and Thomas’ theory of access [35], there are five dimensions that decides the fit between the patient and the healthcare system, namely, 1) availability, 2) accessibility, 3) accommodation, 4) affordability, and 5) acceptance. The authors argue that these factors decide the patients’ likelihood of accessing healthcare services, and hence, one might lack access to care due to challenges overcoming one or more of these dimensions. Therefore, dental programs that aim to tackle those barriers to enhance patients’ ability to access care are more likely to report favorable outcomes. We found that the common mechanism of access utilized by the included studies was by improving patients’ affordability (*n* = 17, 68%). This was achieved by either providing free dental services to those who might have been facing financial challenges or by expanding the eligibility criteria of various already existing governmental programs (e.g., Medicaid or Medicare in the United States). The demographics of those included consisted of low-income individuals, people enrolled in social assistance programs, and those experiencing homelessness.

In one study, the removal of user fees for primary dental care services in a community with a high poverty rate in South Africa (at any age group) significantly increased patient attendance in dental settings [39]. In contrast, access to cost-free preventive treatment (for children with unmet dental needs) did not show the same results in the US, and the challenges associated with utilization varied between urban and rural regions [47]. Sanjeevan et al. [56], in their systematic review, observed similar findings indicating the impact of access on promoting successful change is dependent on different contexts (population types and political environment) in which it is provided.

Other studies addressed availability, accessibility, and acceptance by providing care to vulnerable individuals who had experiences with discrimination, racism and geographic isolation. For instance, Metsch and colleagues [49] provided referral services to individuals diagnosed with HIV who have experience a history of discrimination that prohibited them from accessing the required dental care. In addition, DiMarco et al. [40] and Rozier et al. [55] increased the availability and acceptability of services by training medical personnel to provide referral and preventive dental services to children unable to access dental care. Finally, we identified one study [55] that accommodated for the patients’ needs by providing the planned dental services during the children’s routine medical visits.

## Discussion

Despite the well-documented consequences of unaddressed dental problems, we found minimal evidence around impacts of addressing dental problems. Our search strategy identified 25 studies that examined the impacts of providing dental care programs from the broad societal and healthcare system perspectives. The healthcare outcomes identified were the utilization of services, expenditure and cost-savings, and the number of averted treatments. While the societal outcomes identified were employability and homelessness.

While we found some evidence to suggest that implementing dental programs can reduce medical and dental healthcare utilization (especially for non-preventive services) and avert future more invasive treatments, the quantity and quality of studies examining the impacts of dental programs on the future expenditure and cost savings produced yielded weaker and inconclusive conclusions. As for societal outcomes, very limited evidence has been identified about the impact of dental programs on employability and homelessness. One study addressed the impact of providing shelter-based dental programs on the status of veterans (adults and elders) experiencing homelessness. The authors found that even after adjusting for non-dental care variables, those who received dental care through the national veteran rehabilitation program were more likely to secure permanent housing and become more financially stable compared to those who did not receive dental care. The other societal outcomes captured was employability. In the systematic review conducted by Singhal et al. [11], the authors found very little evidence to support the claim that dental care can improve employability outcomes. A few years later, Singhal et al. conducted a retrospective analysis of administrative data retrieved from five regions in Ontario, Canada, related to employment outcomes from the province’s Ministry of Community and Social Services [58]. The purpose was to assess whether employment outcome would differ between those who received dental care through the province’s social assistant dental program and those who did not. The authors found that after one year, the impacts of dental care were not significantly associated with leaving the social assistance programs; however, there was an equity impact. Not everyone on social assistance was disadvantaged in a similar way, people who had denture needs were found to be more dependent on social assistance program compared to those who had preventive needs; however, over the time, denture recipients started leaving social assistance at a similar rate as those who received preventive services. In addition, it is important to consider that some societal impacts such as changes in employability outcomes might take years before any tangible differences can be detected.

Diagnostic and preventive programs are by far the most popular dental program. Likely, because of their lower cost and relative higher financial sustainability. However, despite the inclusion of high-quality studies only (4 + stars), the lack of clarity about the provided services remains a significant reporting concern. Moreover, we only identified three studies that assessed the impacts of “comprehensive dental programs”, therefore, it is challenging to ascertain the impacts of interventional dental care on the examined outcomes.

In the American context, where dental care is highly privatized, the inability to access dental services due to cost-related issues remain the most significant barrier. Therefore, the majority of identified programs aim to tackle affordability by providing free dental care through Medicaid and state-sponsored programs. However, it is important to acknowledge the other forms of barriers that prohibit access to dental care. Therefore, increasing availability of dental care services by training medical personnel and addressing the stigma associated with HIV were important mechanisms of access addressed by some studies but were not as popular as tackling affordability issues. Meanwhile, a significant number of studies did not specify the mechanism by which their program address the unique barriers to dental care, preventing us from making inferences about the success of those interventions.

The overall majority of identified programs have demonstrated success in 1) reducing the utilization healthcare services for dental problems and increasing the utilization of preventive dental care services, 2) averting future invasive treatments, and to a lesser extent, 3) demonstrating cost-saving effects/reduction in expenditure. However, due to the small number of studies identified, and the significant heterogeneity of the programs included (i.e., differences in the context of dental care, funding status, etc.), it is not possible to ascertain attribution of programs to the changes identified. Nevertheless, it highlights the success of programs in reducing some of the societal and economic burden of dental diseases.

Although being only a few, it was commendable to find studies that address important societal outcomes such as homelessness and employability. However, given the significant burden of dental diseases have on societies, more emphasis must be put on examining the impacts of dental programs on other societal outcomes. This includes but not limited to school attendance, academic performances, economic productivities (presenteeism and absenteeism), quality of life, and social interactions.

As for the strengths of this review, this is the first study to examine the impacts of providing dental care from the healthcare and societal levels. It also underscores the paucity of studies addressing the potential benefits of implementing dental care programs. On the other hand, a number of limitations have to be considered when interpreting the results from this scoping review. First, we included the impacts of dental care at healthcare and societal levels, not individual and family level outcomes. All outcome levels are important but including all aspects in one review can be confusing. Therefore, we decided to focus only on the broader impacts (health care and societal levels of dental care programs). Second, we included articles only in English language, and hence we might have missed articles that have been conducted and published from non-English-speaking countries. Third, direct comparison across all types of dental care interventions was not possible due to differences in the contexts, the wide variety in populations that interventions targeted, and the included studies’ design. Finally, we did not include an analysis of the follow-up intervals, and as such, we cannot comment on the long-term effectiveness and sustainability of these programs. It is worth mentioning that most interventions targeted at least two mechanisms. They continually and unpredictably interact with other elements over time as a complex system, and therefore, the degree of overlap between them to produce the observed outcome is unclear. Nonetheless, we believe that the findings from this review will help guide policymakers about the types of dental care program to allocate resources towards .

## Conclusion

Our study highlights the knowledge gaps in the literature in terms of the scope and outcomes examined when assessing the impacts of dental care programs. In order to recommend sustainable policy solution, studies investigating the impacts of dental care interventions at the broader societal level require more attention in future research.

## Supplementary information

**Additional file 1. **Search Strategy.

**Additional file 2. **Quality assessment tool.

**Additional file 3. **Outcome effectiveness.

**Additional file 4. **Preferred Reporting Items for Systematic reviews and Meta-Analyses extension for Scoping Reviews (PRISMA-ScR) Checklist.

## Data Availability

All data generated or analyzed during this study are included in this published article and its supplementary information files.
