# Supplementary material for: The impact of dental care programs on healthcare system and societal outcomes: a scoping review
Source: BMC Health Serv Res. 2022 Dec 23;22:1574. doi: 10.1186/s12913-022-08951-x (PMC9780625; doi:10.1186/s12913-022-08951-x)
Supplement: Supplementary file 1 — Additional file 1. Search Strategy. [file 12913_2022_8951_MOESM1_ESM.docx]

**Additional file 1 – Search Strategy**

**SEARCH STRATEGY:**

The following databases were searched: MEDLINE, EMBASE, CINAHL and Social Science Abstracts. MEDLINE and EMBASE were searched using the Ovid interface. CINAHL was searched using the EBSCO interface. Social Science Abstracts was searched using the ProQuest interface. The MEDLINE search strategy was developed, and peer reviewed using the PRESS standard. The MEDLINE search was then adapted for the other databases.

Searches in MEDLINE, EMBASE and CINAHL were limited by study design in adherence to the scope of this review. Since Social Science Abstracts is a subject specific database, we only searched the terms for the "Intervention" and did not search the terms for the "outcome." Please see below for the detailed inclusion and exclusion criteria for each database. When no appropriate filters needed are available through the databases searched, external pre-tested and published filters were identified by using the suggested filters from InterTASC Information Specialists’ Sub-Group Search Filter Resource^[[1]](#footnote-1)^. In Ovid MEDLINE, Qualitative studies, Case-control studies and Cohort studies filters were adapted from University of Texas Health Science Center at Houston^[[2]](#footnote-2)^. In Ovid EMBASE, the Observational Studies filter was adapted from Ovid Expert Searches^[[3]](#footnote-3)^. In CINAHL, search filters were adapted from Wilcaynski's article^[[4]](#footnote-4)^. Animal studies were excluded by using the animal filters available and key word searches in each database. Keywords searches were also used to exclude equipment or supplies or dental students or dental schools which are not within the scope of this review.

For the search in Aug. 2019, in total, 28495 records were identified through the database search and imported into a citation management system (EndNote). After de-duplicating with EndNote's default "Duplicates" setting, there were 21570 records for screening. In Jan. 21, 2020, searches were re-run in each database and 2858 results were imported into EndNote. This time, duplicated references were identified and removed using the systematic de-duplication method described by Bramer et al. (2016)^[[5]](#footnote-5)^, resulting in 24117 records for screening (including grey literature and reference check). For the February 2022 search, 7352 records were identified, and after de-duplication, 5388 unique articles were retrieved.

**Ovid MEDLINE: Epub Ahead of Print, In-Process & Other Non-Indexed Citations, Ovid MEDLINE® Daily and Ovid MEDLINE® 1946-Present**

**Language:** English

**Subjects:** Human

**Age group:** No limitation

**Publication year:** after 1999

**Publication Types:** Observational Studies, Systematic Reviews, Meta-analysis

**External Filters used:** qualitative studies, cohort studies, case control studies.

**Exclusion**: equipment and supplies, all animal studies, dental students, dental schools

**Searched on Aug.12, 2019, Jan.21, 2020, and Feb. 19, 2022**

**Records retrieved:** 11255 (Aug. 2019); 2037 (Jan. 2020); 3035 (Feb. 19)

Ovid MEDLINE: Epub Ahead of Print, In-Process & Other Non-Indexed Citations, Ovid MEDLINE® Daily and Ovid MEDLINE® <1946-Present>

| Search history sorted by search number ascending | | | |  |  |  |
| --- | --- | --- | --- | --- | --- | --- |
| # | Searches |  |  |  |  |  |
|  | | | | | | |
| 1 | exp Dental Care/ |  |  |  |  |  |
| 2 | Comprehensive Dental Care/ |  |  |  |  |  |
| 3 | exp Dental clinics/ |  |  |  |  |  |
| 4 | public health dentistry/ or community dentistry/ or fluoridation/ or health education, dental/ |  |  |  |  |  |
| 5 | exp Mouth rehabilitation/ |  |  |  |  |  |
| 6 | exp Oral diagnosis/ |  |  |  |  |  |
| 7 | exp General practice, dental/ |  |  |  |  |  |
| 8 | exp Education, Dental/ |  |  |  |  |  |
| 9 | exp Dental prophylaxis/ |  |  |  |  |  |
| 10 | exp Dental health services/ |  |  |  |  |  |
| 11 | exp Dentistry, Operative/ |  |  |  |  |  |
| 12 | exp Endodontics/ |  |  |  |  |  |
| 13 | exp Esthetics, Dental/ |  |  |  |  |  |
| 14 | exp Infection control, Dental/ |  |  |  |  |  |
| 15 | exp Oral surgical procedures/ |  |  |  |  |  |
| 16 | exp Orthodontics/ |  |  |  |  |  |
| 17 | exp Oral pathology/ |  |  |  |  |  |
| 18 | exp Periodontics/ |  |  |  |  |  |
| 19 | exp Preventive dentistry/ |  |  |  |  |  |
| 20 | exp Prosthodontics/ |  |  |  |  |  |
| 21 | exp Oral surgery/ |  |  |  |  |  |
| 22 | exp Oral medicine/ |  |  |  |  |  |
| 23 | (care adj3 dental).tw,kf. |  |  |  |  |  |
| 24 | (dental adj3 service*).tw,kf. |  |  |  |  |  |
| 25 | (dent* adj2 (check-up or health or intervention* or clinic* or treatment* or therap* or program* or practice* or education or procedure* or restorat* regenerative*)).tw,kf. |  |  |  |  |  |
| 26 | dental stress analys?s.tw,kf. |  |  |  |  |  |
| 27 | denture*.tw,kf. |  |  |  |  |  |
| 28 | (oral adj2 care).tw,kf. |  |  |  |  |  |
| 29 | (dental health adj2 survey*).tw,kf. |  |  |  |  |  |
| 30 | ((decayed or missing or filled) adj3 teeth).tw,kf. |  |  |  |  |  |
| 31 | (bleeding on probing adj3 gingival).tw,kf. |  |  |  |  |  |
| 32 | ((periodont* or orthodontic* or prosthodont* or endodontic) adj2 (treatment* or procedure* or surger* or care)).tw,kf. |  |  |  |  |  |
| 33 | ((dental or t??th or periodontal) adj1 cleaning).tw,kf. |  |  |  |  |  |
| 34 | (endodontics or orthodontics or periodontics or prosthodontics).tw,kf. |  |  |  |  |  |
| 35 | (orthodontia or orthodontolog* or orthodonty).tw,kf. |  |  |  |  |  |
| 36 | (Orthodontic adj2 (space closure or anchorage* or extrusion*)).tw,kf. |  |  |  |  |  |
| 37 | (root canal adj1 (therap* or procedure* or obturation* or preparation* or surger*)).tw,kf. |  |  |  |  |  |
| 38 | ((pulpectom* or pulpotom* or apexification* or apex) adj1 excision*).tw,kf. |  |  |  |  |  |
| 39 | ((oral or mouth) adj2 surg*).tw,kf. |  |  |  |  |  |
| 40 | parotidectom*.tw,kf. |  |  |  |  |  |
| 41 | ((pre prosthetic or preprosthetic) adj1 surger*).tw,kf. |  |  |  |  |  |
| 42 | (cavity lining* adj1 dental).tw,kf. |  |  |  |  |  |
| 43 | (varnish* adj2 cavity).tw,kf. |  |  |  |  |  |
| 44 | (community periodontal index of treatment needs or cpitn).tw,kf. |  |  |  |  |  |
| 45 | ((mouth or oral) adj2 rehabilitation*).tw,kf. |  |  |  |  |  |
| 46 | ((diagnos* or examination*) adj1 (oral or dental)).tw,kf. |  |  |  |  |  |
| 47 | dental caries activity test*.tw,kf. |  |  |  |  |  |
| 48 | (dental adj1 pulp test*).tw,kf. |  |  |  |  |  |
| 49 | ((dental or intraoral or orthodontic) adj1 photograph*).tw,kf. |  |  |  |  |  |
| 50 | ((dental or bitewing or tooth) adj1 (radiography or radiovisiography)).tw,kf. |  |  |  |  |  |
| 51 | (Dental adj1 (x ray or xray)).tw,kf. |  |  |  |  |  |
| 52 | (fluorescence adj1 quantitative light-induced).tw,kf. |  |  |  |  |  |
| 53 | (community adj1 dentistry).tw,kf. |  |  |  |  |  |
| 54 | ((dental or periodontal or t??th) adj1 prophylaxis).tw,kf. |  |  |  |  |  |
| 55 | ((supragingival or subgingival or dental or root) adj1 scaling*).tw,kf. |  |  |  |  |  |
| 56 | ((maxillo-mandibular or maxillomandibular or oral or maxillofacial or orthognathic or jaw) adj1 surg*).tw,kf. |  |  |  |  |  |
| 57 | ((maxillo-mandibular or maxillomandibular or oral or maxillofacial or orthognathic) adj2 procedure*).tw,kf. |  |  |  |  |  |
| 58 | (oral adj1 hygiene).tw,kf. |  |  |  |  |  |
| 59 | ((evidence-based or preventative or cosmetic or operative or prosthetic or reparative) adj2 dentistry).tw,kf. |  |  |  |  |  |
| 60 | (dental adj1 (esthetic* or aesthetic* or floss or scaling* or brace* or hygiene* or polishing or radiology or bonding or soldering* or prosthetic*)).tw,kf. |  |  |  |  |  |
| 61 | ((dental or tooth) adj3 filling*).tw,kf. |  |  |  |  |  |
| 62 | ((dental or tooth or caries) adj2 prevention).tw,kf. |  |  |  |  |  |
| 63 | ((tooth or dental) adj1 (an?esthesia or an?esthetic* or casting*)).tw,kf. |  |  |  |  |  |
| 64 | Jaw relation record*.tw,kf. |  |  |  |  |  |
| 65 | (Sinus adj1 lifting*).tw,kf. |  |  |  |  |  |
| 66 | (dental adj2 (implant* or prosthes?s)).tw,kf. |  |  |  |  |  |
| 67 | ((dental or enamel) adj1 microabrasion*).tw,kf. |  |  |  |  |  |
| 68 | (t??th adj1 (bleaching or whitening or replantation* or reimplantation* or extrusion* or uprighting* or remineralization* or polishing or restoration* or surger* or inlay* or preparation* or removal* or resection*)).tw,kf. |  |  |  |  |  |
| 69 | (Dental adj1 (reimplantation* or replantation* or reinclusion*)).tw,kf. |  |  |  |  |  |
| 70 | (dental adj1 infection adj1 control*).tw,kf. |  |  |  |  |  |
| 71 | (apicoectom* or gingivectom* or gingivoplast* or glossectom*).tw,kf. |  |  |  |  |  |
| 72 | ((tongue or lingual) adj1 (extirpation* or resection*)).tw,kf. |  |  |  |  |  |
| 73 | ((jaw or maxillomandibular) adj1 fixation*).tw,kf. |  |  |  |  |  |
| 74 | (mandibula* adj1 (advancement* or reconstruction* or restoration* or resection*)).tw,kf. |  |  |  |  |  |
| 75 | (mandible ostectom* or mandibulectom*).tw,kf. |  |  |  |  |  |
| 76 | ((maxillofacial or mandibular) adj1 (prosthes?s or impant*)).tw,kf. |  |  |  |  |  |
| 77 | ((upper jaw or maxilla*) adj1 resection).tw,kf. |  |  |  |  |  |
| 78 | maxillectom*.tw,kf. |  |  |  |  |  |
| 79 | genioplast*.tw,kf. |  |  |  |  |  |
| 80 | (chin adj2 (correction* or reconstruction * or reduction* or surger* or operation*)).tw,kf. |  |  |  |  |  |
| 81 | (chinplast* or mentoplast*).tw,kf. |  |  |  |  |  |
| 82 | ((maxillary or mandibular or alveolar) adj1 ridge augmentation*).tw,kf. |  |  |  |  |  |
| 83 | (alveolectom* or alveoloplast* or vestibuloplast*).tw,kf. |  |  |  |  |  |
| 84 | ((endosseous or subperiosteal) adj2 implant*).tw,kf. |  |  |  |  |  |
| 85 | (alveolar adj2 graft*).tw,kf. |  |  |  |  |  |
| 86 | ((le fort or lefort or maxilla* or jaw or mandib*) adj2 osteotom*).tw,kf. |  |  |  |  |  |
| 87 | (Le fort adj1 operation*).tw,kf. |  |  |  |  |  |
| 88 | (sagittal split adj2 osteotom*).tw,kf. |  |  |  |  |  |
| 89 | (sinus adj2 augmentation*).tw,kf. |  |  |  |  |  |
| 90 | ((t??th or serial) adj1 extraction*).tw,kf. |  |  |  |  |  |
| 91 | Exodont*.tw,kf. |  |  |  |  |  |
| 92 | (Molar adj1 (amputation* or extraction*)).tw,kf. |  |  |  |  |  |
| 93 | odontectom*.tw,kf. |  |  |  |  |  |
| 94 | (uvulopharyngopalatoplast* or uvulopalatoplast* or palatouvulopharyngoplast* or pharyngouvulopalatoplast*).tw,kf. |  |  |  |  |  |
| 95 | (dental adj1 (internal or marginal) adj1 adaptation*).tw,kf. |  |  |  |  |  |
| 96 | (dental adj1 internal adj1 fit*).tw,kf. |  |  |  |  |  |
| 97 | (mandibular adj1 advancement).tw,kf. |  |  |  |  |  |
| 98 | (occlusal adj1 (splint* or adjustment* or equilibration*)).tw,kf. |  |  |  |  |  |
| 99 | ((fixed or activator or extraoral) adj2 appliance*).tw,kf. |  |  |  |  |  |
| 100 | ((fixed or bonded or permanent) adj1 retainer*).tw,kf. |  |  |  |  |  |
| 101 | ((clear aligner or herbst or bimler or andresen) adj1 appliance*).tw,kf. |  |  |  |  |  |
| 102 | (frankel adj2 regulator*).tw,kf. |  |  |  |  |  |
| 103 | kinetor*.tw,kf. |  |  |  |  |  |
| 104 | ((function or harvold) adj1 activator*).tw,kf. |  |  |  |  |  |
| 105 | (jasper adj1 jumper*).tw,kf. |  |  |  |  |  |
| 106 | bionator*.tw,kf. |  |  |  |  |  |
| 107 | invisalign*.tw,kf. |  |  |  |  |  |
| 108 | (crown adj1 lengthening).tw,kf. |  |  |  |  |  |
| 109 | (forced adj2 eruption*).tw,kf. |  |  |  |  |  |
| 110 | ((maxillary or palatal) adj1 expansion*).tw,kf. |  |  |  |  |  |
| 111 | palatoplast*.tw,kf. |  |  |  |  |  |
| 112 | (palate adj1 (plast* or reconstruction* or operation*)).tw,kf. |  |  |  |  |  |
| 113 | (tooth adj2 (movement* or intrusion* or depression* or care)).tw,kf. |  |  |  |  |  |
| 114 | ((oral or maxillofacial) adj3 patholog*).tw,kf. |  |  |  |  |  |
| 115 | (periodontal adj1 (medicine*or prosthes?s or splint* or dressing* or prevention*)).tw,kf. |  |  |  |  |  |
| 116 | (periodontal adj2 (tissue regeneration* or debridement*)).tw,kf. |  |  |  |  |  |
| 117 | ((dental or mouth or t??th) adj1 debridement*).tw,kf. |  |  |  |  |  |
| 118 | (root adj1 planing*).tw,kf. |  |  |  |  |  |
| 119 | ((subgingival or gingival) adj1 (curettage* or retraction*)).tw,kf. |  |  |  |  |  |
| 120 | fluoridation*.tw,kf. |  |  |  |  |  |
| 121 | (mouth adj1 (protect* or guard)).tw,kf. |  |  |  |  |  |
| 122 | stomatolog*.tw,kf. |  |  |  |  |  |
| 123 | (medicine adj1 oral).tw,kf. |  |  |  |  |  |
| 124 | or/1-123 |  |  |  |  |  |
| 125 | exp Health promotion/ or Public health/ or exp Patient education as topic/ |  |  |  |  |  |
| 126 | ((promotion* or campaign* or program*) adj2 health).tw,kf. |  |  |  |  |  |
| 127 | (program* adj2 wellness).tw,kf. |  |  |  |  |  |
| 128 | (head start adj1 program*).tw,kf. |  |  |  |  |  |
| 129 | (Pathology or pathological or telepathology).tw,kf. |  |  |  |  |  |
| 130 | ((public or deliver* or system*) adj1 (health care or healthcare)).tw,kf. |  |  |  |  |  |
| 131 | or/125-130 |  |  |  |  |  |
| 132 | limit 131 to dentistry journals |  |  |  |  |  |
| 133 | exp Dentistry/ |  |  |  |  |  |
| 134 | 131 and 133 |  |  |  |  |  |
| 135 | 132 or 134 |  |  |  |  |  |
| 136 | 124 or 135 |  |  |  |  |  |
| 137 | exp Health status/ |  |  |  |  |  |
| 138 | exp Drug utilization/ |  |  |  |  |  |
| 139 | Health Services/ |  |  |  |  |  |
| 140 | exp Community Health Services/ |  |  |  |  |  |
| 141 | exp Hospitalization/ |  |  |  |  |  |
| 142 | exp Emergency Medical Services/ |  |  |  |  |  |
| 143 | Rehabilitation/ |  |  |  |  |  |
| 144 | exp Activities of Daily Living/ |  |  |  |  |  |
| 145 | exp Self care/ |  |  |  |  |  |
| 146 | exp Self management/ |  |  |  |  |  |
| 147 | exp Role/ |  |  |  |  |  |
| 148 | exp Job satisfaction/ |  |  |  |  |  |
| 149 | exp Employment/ |  |  |  |  |  |
| 150 | exp Health resources/ |  |  |  |  |  |
| 151 | exp Economics/ |  |  |  |  |  |
| 152 | exp Economic models/ |  |  |  |  |  |
| 153 | exp Social support/ |  |  |  |  |  |
| 154 | exp Leisure Activity/ |  |  |  |  |  |
| 155 | exp Efficiency/ |  |  |  |  |  |
| 156 | Absenteeism/ |  |  |  |  |  |
| 157 | Presenteeism/ |  |  |  |  |  |
| 158 | Social behavior/ |  |  |  |  |  |
| 159 | exp Social adjustment/ |  |  |  |  |  |
| 160 | exp Social skills/ |  |  |  |  |  |
| 161 | exp Rejection/ |  |  |  |  |  |
| 162 | (health status adj1 (index* or indicator* or indices)).tw,kf. |  |  |  |  |  |
| 163 | (social adj1 (breakdown syndrome* or isolation* or separation* or alienation* or estrangement* or deprivation*)).tw,kf. |  |  |  |  |  |
| 164 | (social adj3 us*).tw,kf. |  |  |  |  |  |
| 165 | responsiveness.tw,kf. |  |  |  |  |  |
| 166 | (drug adj3 utilization).tw,kf. |  |  |  |  |  |
| 167 | ((drug or medicine) adj2 (us* or misus* or consumption*)).tw,kf. |  |  |  |  |  |
| 168 | (health adj3 system*).tw,kf. |  |  |  |  |  |
| 169 | (health adj2 (service* or agenc* or practice*)).tw,kf. |  |  |  |  |  |
| 170 | ((preventative or physician* or diagnostic) adj2 service*).tw,kf. |  |  |  |  |  |
| 171 | (medical adj2 overus*).tw,kf. |  |  |  |  |  |
| 172 | (rehabilitation* or readaptation* or readjustment* or recover* or resociali?ation* or revalidation*).tw,kf. |  |  |  |  |  |
| 173 | (functional adj1 (assessment* or training*)).tw,kf. |  |  |  |  |  |
| 174 | (community adj2 (health or care)).tw,kf. |  |  |  |  |  |
| 175 | (community adj1 (integration* or reintegration* or program*)).tw,kf. |  |  |  |  |  |
| 176 | (daycare adj1 (center* or centre*)).tw,kf. |  |  |  |  |  |
| 177 | ((public health or community) adj2 nursing).tw,kf. |  |  |  |  |  |
| 178 | (assertive adj1 community adj1 treatment*).tw,kf. |  |  |  |  |  |
| 179 | (community adj1 network*).tw,kf. |  |  |  |  |  |
| 180 | (community adj1 pharmac* adj1 service*).tw,kf. |  |  |  |  |  |
| 181 | (family adj1 planning).tw,kf. |  |  |  |  |  |
| 182 | (planned adj1 (pregnanc* or parenthood)).tw,kf. |  |  |  |  |  |
| 183 | ((foster or kinship) adj2 care).tw,kf. |  |  |  |  |  |
| 184 | ((home or domiciliary) adj1 care).tw,kf. |  |  |  |  |  |
| 185 | Hospice*.tw,kf. |  |  |  |  |  |
| 186 | (Maternal adj1 health adj1 service*).tw,kf. |  |  |  |  |  |
| 187 | ((employment or occupational or employee*) adj3 (health or assistance)).tw,kf. |  |  |  |  |  |
| 188 | ((emergency or evacuation) adj1 shelter*).tw,kf. |  |  |  |  |  |
| 189 | ((displaced person* or refugee) adj1 (camp* or settlement* or shelter*)).tw,kf. |  |  |  |  |  |
| 190 | ((senior or aged or elderly) adj3 (centre* or center* or home* or facilit*)).tw,kf. |  |  |  |  |  |
| 191 | Uncompensated care.tw,kf. |  |  |  |  |  |
| 192 | ((work or job or vocation* or life) adj3 (performance* or stress*)).tw,kf. |  |  |  |  |  |
| 193 | satisfaction.tw,kf. |  |  |  |  |  |
| 194 | employment.tw,kf. |  |  |  |  |  |
| 195 | labo?r force.tw,kf. |  |  |  |  |  |
| 196 | (occupational adj2 status).tw,kf. |  |  |  |  |  |
| 197 | underemployment.tw,kf. |  |  |  |  |  |
| 198 | ((career or clinical or job) adj1 ladder*).tw,kf. |  |  |  |  |  |
| 199 | (career adj1 mobilit*).tw,kf. |  |  |  |  |  |
| 200 | ((back or return) adj2 work).tw,kf. |  |  |  |  |  |
| 201 | unemployment.tw,kf. |  |  |  |  |  |
| 202 | (health adj1 (resource* or workforce)).tw,kf. |  |  |  |  |  |
| 203 | (health adj2 manpower).tw,kf. |  |  |  |  |  |
| 204 | hospitali?ation*.tw,kf. |  |  |  |  |  |
| 205 | (hospital adj2 service*).tw,kf. |  |  |  |  |  |
| 206 | hospital stay*.tw,kf. |  |  |  |  |  |
| 207 | (length* adj2 stay*).tw,kf. |  |  |  |  |  |
| 208 | ((patient or voluntary) adj1 admission*).tw,kf. |  |  |  |  |  |
| 209 | ((hospital or patient) adj1 readmission*).tw,kf. |  |  |  |  |  |
| 210 | economic*.tw,kf. |  |  |  |  |  |
| 211 | ((macroeconomic or microeconomic or socioeconomic) adj1 (factor* or aspect*)).tw,kf. |  |  |  |  |  |
| 212 | (income adj2 (group* or level* or classification* or high* or middle or low*)).tw,kf. |  |  |  |  |  |
| 213 | (educational adj2 status*).tw,kf. |  |  |  |  |  |
| 214 | literac*.tw,kf. |  |  |  |  |  |
| 215 | (consumer price adj1 (index* or indices)).tw,kf. |  |  |  |  |  |
| 216 | (cost* adj2 living).tw,kf. |  |  |  |  |  |
| 217 | household consumption*.tw,kf. |  |  |  |  |  |
| 218 | (utility adj1 theor*).tw,kf. |  |  |  |  |  |
| 219 | compensation*.tw,kf. |  |  |  |  |  |
| 220 | (resource adj2 allocation*).tw,kf. |  |  |  |  |  |
| 221 | (allocative adj1 efficiency).tw,kf. |  |  |  |  |  |
| 222 | ((healthcare or health care) adj1 ration*).tw,kf. |  |  |  |  |  |
| 223 | expropriation*.tw,kf. |  |  |  |  |  |
| 224 | (marginal adj1 analys?s).tw,kf. |  |  |  |  |  |
| 225 | (cost* adj2 (analys?s or benefit*)).tw,kf. |  |  |  |  |  |
| 226 | (cost* adj1 (effectiveness or comparison* or measure* or allocation* or apportionment* or shifting* or containment* or control* or sharing or saving*)).tw,kf. |  |  |  |  |  |
| 227 | (out of pocket adj1 (cost* or pay* or spending)).tw,kf. |  |  |  |  |  |
| 228 | (expenditure* or expense*).tw,kf. |  |  |  |  |  |
| 229 | (return on investment or roi).tw,kf. |  |  |  |  |  |
| 230 | affordabilit*.tw,kf. |  |  |  |  |  |
| 231 | pricing.tw,kf. |  |  |  |  |  |
| 232 | ((medical or health* or treatment or drug or hospital) adj2 cost*).tw,kf. |  |  |  |  |  |
| 233 | (direct service adj1 cost*).tw,kf. |  |  |  |  |  |
| 234 | (charge* or fee or fees).tw,kf. |  |  |  |  |  |
| 235 | (rate set* adj2 review).tw,kf. |  |  |  |  |  |
| 236 | ((group or shared or joint) adj1 purchasing).tw,kf. |  |  |  |  |  |
| 237 | (health adj2 marketing).tw,kf. |  |  |  |  |  |
| 238 | ((healthcare or health care) adj1 (industr* or market* or sector*)).tw,kf. |  |  |  |  |  |
| 239 | investment*.tw,kf. |  |  |  |  |  |
| 240 | (federal adj2 aid*).tw,kf. |  |  |  |  |  |
| 241 | social welfare.tw,kf. |  |  |  |  |  |
| 242 | (social adj1 (security or insurance)).tw,kf. |  |  |  |  |  |
| 243 | (aid adj3 families with dependent children).tw,kf. |  |  |  |  |  |
| 244 | (family adj1 allowance*).tw,kf. |  |  |  |  |  |
| 245 | ((psychological or psychosocial or social) adj1 support*).tw,kf. |  |  |  |  |  |
| 246 | (coping adj2 (behavio?r* or abilit* or strateg*)).tw,kf. |  |  |  |  |  |
| 247 | ((behavio?r* or abilit* or competenc* or skill*) adj1 social).tw,kf. |  |  |  |  |  |
| 248 | ((psychological or emotional or personal) adj adjustment*).tw,kf. |  |  |  |  |  |
| 249 | (emotional adj (adaptation or equilibrium)).tw,kf. |  |  |  |  |  |
| 250 | (behavio?r adj1 (permissive or development or facilitation or pattern or variable)).tw,kf. |  |  |  |  |  |
| 251 | (behavio?r* adj (variable or activity or characteristic or response or specificity or symptoms)).tw,kf. |  |  |  |  |  |
| 252 | (attitude adj2 (health or illness or sickness or patient* or dental or dentist*)).tw,kf. |  |  |  |  |  |
| 253 | (psychological adj3 (phenomena or well* or well-being)).tw,kf. |  |  |  |  |  |
| 254 | (interpersonal adj1 (skill* or relation* or communication*)).tw,kf. |  |  |  |  |  |
| 255 | (shy* or timid* or assertiveness).tw,kf. |  |  |  |  |  |
| 256 | (rejection* or permissiveness).tw,kf. |  |  |  |  |  |
| 257 | leisure*.tw,kf. |  |  |  |  |  |
| 258 | (festival* or holiday*).tw,kf. |  |  |  |  |  |
| 259 | recreation*.tw,kf. |  |  |  |  |  |
| 260 | relaxation*.tw,kf. |  |  |  |  |  |
| 261 | (hobby or hobbies).tw,kf. |  |  |  |  |  |
| 262 | (sport* or athletic*).tw,kf. |  |  |  |  |  |
| 263 | efficiency.tw,kf. |  |  |  |  |  |
| 264 | productivity.tw,kf. |  |  |  |  |  |
| 265 | ((accident* or emergenc*) adj2 service*).tw,kf. |  |  |  |  |  |
| 266 | (emergency adj2 care).tw,kf. |  |  |  |  |  |
| 267 | emergicenter*.tw,kf. |  |  |  |  |  |
| 268 | advanced trauma life support.tw,kf. |  |  |  |  |  |
| 269 | (emergency adj2 dispatch*).tw,kf. |  |  |  |  |  |
| 270 | (ems adj1 communication adj1 system*).tw,kf. |  |  |  |  |  |
| 271 | (emergency adj1 hospital adj1 service*).tw,kf. |  |  |  |  |  |
| 272 | (emergency adj1 (ward* or department* or room* or centre* or center* or dispensar*)).tw,kf. |  |  |  |  |  |
| 273 | (trauma adj1 (center* or centre*)).tw,kf. |  |  |  |  |  |
| 274 | (emergency adj2 unit*).tw,kf. |  |  |  |  |  |
| 275 | (emergency adj3 (visit* or admission*)).tw,kf. |  |  |  |  |  |
| 276 | (emergency adj1 psychiatric adj1 service*).tw,kf. |  |  |  |  |  |
| 277 | (patient* adj2 transport*).tw,kf. |  |  |  |  |  |
| 278 | (wounded adj2 sick adj2 transport*).tw,kf. |  |  |  |  |  |
| 279 | triage*.tw,kf. |  |  |  |  |  |
| 280 | (organizational adj1 involvement*).tw,kf. |  |  |  |  |  |
| 281 | (health adj3 indicator*).tw,kf. |  |  |  |  |  |
| 282 | (time adj4 loss).tw,kf. |  |  |  |  |  |
| 283 | or/137-282 |  |  |  |  |  |
| 284 | 136 and 283 |  |  |  |  |  |
| 285 | limit 284 to animals |  |  |  |  |  |
| 286 | 284 not 285 |  |  |  |  |  |
| 287 | limit 286 to (english language and yr="1999 -Current" and journal article) |  |  |  |  |  |
| 288 | limit 287 to (meta analysis or observational study or "systematic review") |  |  |  |  |  |
| 289 | ((("semi-structured" or semistructured or unstructured or informal or "in-depth" or indepth or "face-to-face" or structured or guide) adj3 (discussion* or questionnaire*)) or (focus group* or interview* or qualitative or ethnograph* or fieldwork or "field work" or "key informant")).ti,ab. or interviews as topic/ or focus groups/ or narration/ or qualitative research/ |  |  |  |  |  |
| 290 | Case-Control Studies/ or Control Groups/ or Matched-Pair Analysis/ or ((case* adj5 control*) or (case adj3 comparison*) or control group*).ti,ab. |  |  |  |  |  |
| 291 | cohort studies/ or longitudinal studies/ or follow-up studies/ or prospective studies/ or retrospective studies/ or cohort.ti,ab. or longitudinal.ti,ab. or prospective.ti,ab. or retrospective.ti,ab. |  |  |  |  |  |
| 292 | 289 or 290 or 291 |  |  |  |  |  |
| 293 | 287 and 292 |  |  |  |  |  |
| 294 | 288 or 293 |  |  |  |  |  |
| 295 | (rats or rat or mice or dog* or pig* or horse* or dog* or mouse or rabbit* or Animal*).tw,kw. |  |  |  |  |  |
| 296 | (Equipment* or instrument* or device* or supply or supplies or t??th brush* or t??thbrush or tooth paste or toothpaste or versus).ti. |  |  |  |  |  |
| 297 | (dental adj2 (student* or school* or university or universities or college*)).tw,kf. |  |  |  |  |  |
| 298 | 295 or 296 or 297 |  |  |  |  |  |
| 299 | 294 not 298 |  |  |  |  |  |

**Key**

/ = indexing term (MeSH heading)

exp = exploded indexing term (MeSH heading)

* = truncation

? = wildcard symbol as a substitute for one character or none

tw = text word search in title or abstract fields

kf = terms in author provided keyword

pt = publication type

adjn = terms within (n-1) words of each other (any order)

**EMBASE:**

**Language:** English

**Subjects:** Not animals

**Age group:** No limitation

**Publication year:** after 1999

**Clinical Queries**: "qualitative (best balance of sensitivity and specificity)"

**External Filters used**: cohort studies, case control studies

**EBM-Evidence Based Medicine:** all

**Exclusion**: equipment and supplies, all animal studies, dental students, dental schools

**Searched on Aug.12, 2019, Jan.21, 2020, Feb 19. 2022**

**Records retrieved: 11498 (Aug. 2019); 745 (Jan. 2020); 1792 (Feb. 2022)**

**Embase Classic+Embase**

| **#** | **Searches** |  |  |  |  |  |
| --- | --- | --- | --- | --- | --- | --- |
| 1 | exp dental procedure/ |  |  |  |  |  |
| 2 | dental clinics/ |  |  |  |  |  |
| 3 | dental health education/ |  |  |  |  |  |
| 4 | exp Dental prophylaxis/ |  |  |  |  |  |
| 5 | operative dentistry/ |  |  |  |  |  |
| 6 | Endodontics/ |  |  |  |  |  |
| 7 | Orthodontics/ |  |  |  |  |  |
| 8 | Periodontics/ |  |  |  |  |  |
| 9 | Preventive dentistry/ |  |  |  |  |  |
| 10 | Prosthodontics/ |  |  |  |  |  |
| 11 | exp Oral surgery/ |  |  |  |  |  |
| 12 | (care adj3 dental).tw,kw. |  |  |  |  |  |
| 13 | (dental adj3 service*).tw,kw. |  |  |  |  |  |
| 14 | (dent* adj2 (check-up or health or intervention* or clinic* or treatment* or therap* or program* or practice* or education or procedure* or restorat* regenerative*)).tw,kw. |  |  |  |  |  |
| 15 | dental stress analys?s.tw,kw. |  |  |  |  |  |
| 16 | denture*.tw,kw. |  |  |  |  |  |
| 17 | (oral adj2 care).tw,kw. |  |  |  |  |  |
| 18 | (dental health adj2 survey*).tw,kw. |  |  |  |  |  |
| 19 | ((decayed or missing or filled) adj3 teeth).tw,kw. |  |  |  |  |  |
| 20 | (bleeding on probing adj3 gingival).tw,kw. |  |  |  |  |  |
| 21 | ((periodont* or orthodontic* or prosthodont* or endodontic) adj2 (treatment* or procedure* or surger* or care)).tw,kw. |  |  |  |  |  |
| 22 | ((dental or t??th or periodontal) adj1 cleaning).tw,kw. |  |  |  |  |  |
| 23 | (endodontics or orthodontics or periodontics or prosthodontics).tw,kw. |  |  |  |  |  |
| 24 | (orthodontia or orthodontolog* or orthodonty).tw,kw. |  |  |  |  |  |
| 25 | (Orthodontic adj2 (space closure or anchorage* or extrusion*)).tw,kw. |  |  |  |  |  |
| 26 | (root canal adj1 (therap* or procedure* or obturation* or preparation* or surger*)).tw,kw. |  |  |  |  |  |
| 27 | ((pulpectom* or pulpotom* or apexification* or apex) adj1 excision*).tw,kw. |  |  |  |  |  |
| 28 | ((oral or mouth) adj2 surg*).tw,kw. |  |  |  |  |  |
| 29 | parotidectom*.tw,kw. |  |  |  |  |  |
| 30 | ((pre prosthetic or preprosthetic) adj1 surger*).tw,kw. |  |  |  |  |  |
| 31 | (cavity lining* adj1 dental).tw,kw. |  |  |  |  |  |
| 32 | (varnish* adj2 cavity).tw,kw. |  |  |  |  |  |
| 33 | (community periodontal index of treatment needs or cpitn).tw,kw. |  |  |  |  |  |
| 34 | ((mouth or oral) adj2 rehabilitation*).tw,kw. |  |  |  |  |  |
| 35 | ((diagnos* or examination*) adj1 (oral or dental)).tw,kw. |  |  |  |  |  |
| 36 | dental caries activity test*.tw,kw. |  |  |  |  |  |
| 37 | (dental adj1 pulp test*).tw,kw. |  |  |  |  |  |
| 38 | ((dental or intraoral or orthodontic) adj1 photograph*).tw,kw. |  |  |  |  |  |
| 39 | ((dental or bitewing or tooth) adj1 (radiography or radiovisiography)).tw,kw. |  |  |  |  |  |
| 40 | (Dental adj1 (x ray or xray)).tw,kw. |  |  |  |  |  |
| 41 | (fluorescence adj1 quantitative light-induced).tw,kw. |  |  |  |  |  |
| 42 | (community adj1 dentistry).tw,kw. |  |  |  |  |  |
| 43 | ((dental or periodontal or t??th) adj1 prophylaxis).tw,kw. |  |  |  |  |  |
| 44 | ((supragingival or subgingival or dental or root) adj1 scaling*).tw,kw. |  |  |  |  |  |
| 45 | ((maxillo-mandibular or maxillomandibular or oral or maxillofacial or orthognathic or jaw) adj1 surg*).tw,kw. |  |  |  |  |  |
| 46 | ((maxillo-mandibular or maxillomandibular or oral or maxillofacial or orthognathic) adj2 procedure*).tw,kw. |  |  |  |  |  |
| 47 | (oral adj1 hygiene).tw,kw. |  |  |  |  |  |
| 48 | ((evidence-based or preventative or cosmetic or operative or prosthetic or reparative) adj2 dentistry).tw,kw. |  |  |  |  |  |
| 49 | (dental adj1 (esthetic* or aesthetic* or floss or scaling* or brace* or hygiene* or polishing or radiology or bonding or soldering* or prosthetic*)).tw,kw. |  |  |  |  |  |
| 50 | ((dental or tooth) adj3 filling*).tw,kw. |  |  |  |  |  |
| 51 | ((dental or tooth or caries) adj2 prevention).tw,kw. |  |  |  |  |  |
| 52 | ((tooth or dental) adj1 (an?esthesia or an?esthetic* or casting*)).tw,kw. |  |  |  |  |  |
| 53 | Jaw relation record*.tw,kw. |  |  |  |  |  |
| 54 | (Sinus adj1 lifting*).tw,kw. |  |  |  |  |  |
| 55 | (dental adj2 (implant* or prosthes?s)).tw,kw. |  |  |  |  |  |
| 56 | ((dental or enamel) adj1 microabrasion*).tw,kw. |  |  |  |  |  |
| 57 | (t??th adj1 (bleaching or whitening or replantation* or reimplantation* or extrusion* or uprighting* or remineralization* or polishing or restoration* or surger* or inlay* or preparation* or removal* or resection*)).tw,kw. |  |  |  |  |  |
| 58 | (Dental adj1 (reimplantation* or replantation* or reinclusion*)).tw,kw. |  |  |  |  |  |
| 59 | (dental adj1 infection adj1 control*).tw,kw. |  |  |  |  |  |
| 60 | (apicoectom* or gingivectom* or gingivoplast* or glossectom*).tw,kw. |  |  |  |  |  |
| 61 | ((tongue or lingual) adj1 (extirpation* or resection*)).tw,kw. |  |  |  |  |  |
| 62 | ((jaw or maxillomandibular) adj1 fixation*).tw,kw. |  |  |  |  |  |
| 63 | (mandibula* adj1 (advancement* or reconstruction* or restoration* or resection*)).tw,kw. |  |  |  |  |  |
| 64 | (mandible ostectom* or mandibulectom*).tw,kw. |  |  |  |  |  |
| 65 | ((maxillofacial or mandibular) adj1 (prosthes?s or impant*)).tw,kw. |  |  |  |  |  |
| 66 | ((upper jaw or maxilla*) adj1 resection).tw,kw. |  |  |  |  |  |
| 67 | maxillectom*.tw,kw. |  |  |  |  |  |
| 68 | genioplast*.tw,kw. |  |  |  |  |  |
| 69 | (chin adj2 (correction* or reconstruction * or reduction* or surger* or operation*)).tw,kw. |  |  |  |  |  |
| 70 | (chinplast* or mentoplast*).tw,kw. |  |  |  |  |  |
| 71 | ((maxillary or mandibular or alveolar) adj1 ridge augmentation*).tw,kw. |  |  |  |  |  |
| 72 | (alveolectom* or alveoloplast* or vestibuloplast*).tw,kw. |  |  |  |  |  |
| 73 | ((endosseous or subperiosteal) adj2 implant*).tw,kw. |  |  |  |  |  |
| 74 | (alveolar adj2 graft*).tw,kw. |  |  |  |  |  |
| 75 | ((le fort or lefort or maxilla* or jaw or mandib*) adj2 osteotom*).tw,kw. |  |  |  |  |  |
| 76 | (Le fort adj1 operation*).tw,kw. |  |  |  |  |  |
| 77 | (sagittal split adj2 osteotom*).tw,kw. |  |  |  |  |  |
| 78 | (sinus adj2 augmentation*).tw,kw. |  |  |  |  |  |
| 79 | ((t??th or serial) adj1 extraction*).tw,kw. |  |  |  |  |  |
| 80 | Exodont*.tw,kw. |  |  |  |  |  |
| 81 | (Molar adj1 (amputation* or extraction*)).tw,kw. |  |  |  |  |  |
| 82 | odontectom*.tw,kw. |  |  |  |  |  |
| 83 | (uvulopharyngopalatoplast* or uvulopalatoplast* or palatouvulopharyngoplast* or pharyngouvulopalatoplast*).tw,kw. |  |  |  |  |  |
| 84 | (dental adj1 (internal or marginal) adj1 adaptation*).tw,kw. |  |  |  |  |  |
| 85 | (dental adj1 internal adj1 fit*).tw,kw. |  |  |  |  |  |
| 86 | (mandibular adj1 advancement).tw,kw. |  |  |  |  |  |
| 87 | (occlusal adj1 (splint* or adjustment* or equilibration*)).tw,kw. |  |  |  |  |  |
| 88 | ((fixed or activator or extraoral) adj2 appliance*).tw,kw. |  |  |  |  |  |
| 89 | ((fixed or bonded or permanent) adj1 retainer*).tw,kw. |  |  |  |  |  |
| 90 | ((clear aligner or herbst or bimler or andresen) adj1 appliance*).tw,kw. |  |  |  |  |  |
| 91 | (frankel adj2 regulator*).tw,kw. |  |  |  |  |  |
| 92 | kinetor*.tw,kw. |  |  |  |  |  |
| 93 | ((function or harvold) adj1 activator*).tw,kw. |  |  |  |  |  |
| 94 | (jasper adj1 jumper*).tw,kw. |  |  |  |  |  |
| 95 | bionator*.tw,kw. |  |  |  |  |  |
| 96 | invisalign*.tw,kw. |  |  |  |  |  |
| 97 | (crown adj1 lengthening).tw,kw. |  |  |  |  |  |
| 98 | (forced adj2 eruption*).tw,kw. |  |  |  |  |  |
| 99 | ((maxillary or palatal) adj1 expansion*).tw,kw. |  |  |  |  |  |
| 100 | palatoplast*.tw,kw. |  |  |  |  |  |
| 101 | (palate adj1 (plast* or reconstruction* or operation*)).tw,kw. |  |  |  |  |  |
| 102 | (tooth adj2 (movement* or intrusion* or depression* or care)).tw,kw. |  |  |  |  |  |
| 103 | ((oral or maxillofacial) adj3 patholog*).tw,kw. |  |  |  |  |  |
| 104 | (periodontal adj1 (medicine*or prosthes?s or splint* or dressing* or prevention*)).tw,kw. |  |  |  |  |  |
| 105 | (periodontal adj2 (tissue regeneration* or debridement*)).tw,kw. |  |  |  |  |  |
| 106 | ((dental or mouth or t??th) adj1 debridement*).tw,kw. |  |  |  |  |  |
| 107 | (root adj1 planing*).tw,kw. |  |  |  |  |  |
| 108 | ((subgingival or gingival) adj1 (curettage* or retraction*)).tw,kw. |  |  |  |  |  |
| 109 | fluoridation*.tw,kw. |  |  |  |  |  |
| 110 | (mouth adj1 (protect* or guard)).tw,kw. |  |  |  |  |  |
| 111 | stomatolog*.tw,kw. |  |  |  |  |  |
| 112 | (medicine adj1 oral).tw,kw. |  |  |  |  |  |
| 113 | or/1-112 |  |  |  |  |  |
| 114 | exp Health promotion/ |  |  |  |  |  |
| 115 | exp Infection control/ |  |  |  |  |  |
| 116 | exp pathology/ |  |  |  |  |  |
| 117 | Public health/ |  |  |  |  |  |
| 118 | ((promotion* or campaign* or program*) adj2 health).tw,kw. |  |  |  |  |  |
| 119 | (program* adj2 wellness).tw,kw. |  |  |  |  |  |
| 120 | (head start adj1 program*).tw,kw. |  |  |  |  |  |
| 121 | (Pathology or pathological or telepathology).tw,kw. |  |  |  |  |  |
| 122 | ((public or deliver* or system*) adj1 (health care or healthcare)).tw,kw. |  |  |  |  |  |
| 123 | or/114-122 |  |  |  |  |  |
| 124 | exp dentistry/ |  |  |  |  |  |
| 125 | 123 and 124 |  |  |  |  |  |
| 126 | 113 or 125 |  |  |  |  |  |
| 127 | exp Health status/ |  |  |  |  |  |
| 128 | Drug utilization/ |  |  |  |  |  |
| 129 | Health Service/ |  |  |  |  |  |
| 130 | Community care/ |  |  |  |  |  |
| 131 | community based rehabilitation/ or community integration/ or emergency shelter/ or community program/ or refugee camp/ or relief work/ or senior center/ |  |  |  |  |  |
| 132 | exp community health nursing/ |  |  |  |  |  |
| 133 | exp preventive health service/ |  |  |  |  |  |
| 134 | Hospitalization/ |  |  |  |  |  |
| 135 | exp emergency health service/ |  |  |  |  |  |
| 136 | Rehabilitation/ |  |  |  |  |  |
| 137 | daily life activity/ |  |  |  |  |  |
| 138 | exp Self care/ |  |  |  |  |  |
| 139 | exp Employment/ |  |  |  |  |  |
| 140 | health care planning/ |  |  |  |  |  |
| 141 | exp Economics/ |  |  |  |  |  |
| 142 | Social support/ |  |  |  |  |  |
| 143 | Social behavior/ |  |  |  |  |  |
| 144 | social interaction/ |  |  |  |  |  |
| 145 | community participation/ |  |  |  |  |  |
| 146 | social competence/ |  |  |  |  |  |
| 147 | social participation/ |  |  |  |  |  |
| 148 | Social disability/ |  |  |  |  |  |
| 149 | Social stigma/ |  |  |  |  |  |
| 150 | Leisure/ |  |  |  |  |  |
| 151 | productivity/ |  |  |  |  |  |
| 152 | [(health status adj1 (index* or indicator* or indices)).tw,kf.] |  |  |  |  |  |
| 153 | (social adj1 (breakdown syndrome* or isolation* or separation* or alienation* or estrangement* or deprivation*)).tw,kw. |  |  |  |  |  |
| 154 | (social adj3 us*).tw,kw. |  |  |  |  |  |
| 155 | responsiveness.tw,kw. |  |  |  |  |  |
| 156 | (drug adj3 utilization).tw,kw. |  |  |  |  |  |
| 157 | ((drug or medicine) adj2 (us* or misus* or consumption*)).tw,kw. |  |  |  |  |  |
| 158 | (health adj3 system*).tw,kw. |  |  |  |  |  |
| 159 | (health adj2 (service* or agenc* or practice*)).tw,kw. |  |  |  |  |  |
| 160 | ((preventative or physician* or diagnostic) adj2 service*).tw,kw. |  |  |  |  |  |
| 161 | (medical adj2 overus*).tw,kw. |  |  |  |  |  |
| 162 | (rehabilitation* or readaptation* or readjustment* or recover* or resociali?ation* or revalidation*).tw,kw. |  |  |  |  |  |
| 163 | (functional adj1 (assessment* or training*)).tw,kw. |  |  |  |  |  |
| 164 | (community adj2 (health or care)).tw,kw. |  |  |  |  |  |
| 165 | (community adj1 (integration* or reintegration* or program*)).tw,kw. |  |  |  |  |  |
| 166 | (daycare adj1 (center* or centre*)).tw,kw. |  |  |  |  |  |
| 167 | ((public health or community) adj2 nursing).tw,kw. |  |  |  |  |  |
| 168 | (assertive adj1 community adj1 treatment*).tw,kw. |  |  |  |  |  |
| 169 | (community adj1 network*).tw,kw. |  |  |  |  |  |
| 170 | (community adj1 pharmac* adj1 service*).tw,kw. |  |  |  |  |  |
| 171 | (family adj1 planning).tw,kw. |  |  |  |  |  |
| 172 | (planned adj1 (pregnanc* or parenthood)).tw,kw. |  |  |  |  |  |
| 173 | ((foster or kinship) adj2 care).tw,kw. |  |  |  |  |  |
| 174 | ((home or domiciliary) adj1 care).tw,kw. |  |  |  |  |  |
| 175 | Hospice*.tw,kw. |  |  |  |  |  |
| 176 | (Maternal adj1 health adj1 service*).tw,kw. |  |  |  |  |  |
| 177 | ((employment or occupational or employee*) adj3 (health or assistance)).tw,kw. |  |  |  |  |  |
| 178 | ((emergency or evacuation) adj1 shelter*).tw,kw. |  |  |  |  |  |
| 179 | ((displaced person* or refugee) adj1 (camp* or settlement* or shelter*)).tw,kw. |  |  |  |  |  |
| 180 | ((senior or aged or elderly) adj3 (centre* or center* or home* or facilit*)).tw,kw. |  |  |  |  |  |
| 181 | Uncompensated care.tw,kw. |  |  |  |  |  |
| 182 | ((work or job or vocation* or life) adj3 (performance* or stress*)).tw,kw. |  |  |  |  |  |
| 183 | satisfaction*.tw,kw. |  |  |  |  |  |
| 184 | employment.tw,kw. |  |  |  |  |  |
| 185 | labo?r force.tw,kw. |  |  |  |  |  |
| 186 | (occupational adj2 status).tw,kw. |  |  |  |  |  |
| 187 | underemployment.tw,kw. |  |  |  |  |  |
| 188 | ((career or clinical or job) adj1 ladder*).tw,kw. |  |  |  |  |  |
| 189 | (career adj1 mobilit*).tw,kw. |  |  |  |  |  |
| 190 | ((back or return) adj2 work).tw,kw. |  |  |  |  |  |
| 191 | unemployment.tw,kw. |  |  |  |  |  |
| 192 | (health adj1 (resource* or workforce)).tw,kw. |  |  |  |  |  |
| 193 | (health adj2 manpower).tw,kw. |  |  |  |  |  |
| 194 | hospitali?ation*.tw,kw. |  |  |  |  |  |
| 195 | (hospital adj2 service*).tw,kw. |  |  |  |  |  |
| 196 | hospital stay*.tw,kw. |  |  |  |  |  |
| 197 | (length* adj2 stay*).tw,kw. |  |  |  |  |  |
| 198 | ((patient or voluntary) adj1 admission*).tw,kw. |  |  |  |  |  |
| 199 | ((hospital or patient) adj1 readmission*).tw,kw. |  |  |  |  |  |
| 200 | economic*.tw,kw. |  |  |  |  |  |
| 201 | ((macroeconomic or microeconomic or socioeconomic) adj1 (factor* or aspect*)).tw,kw. |  |  |  |  |  |
| 202 | (income adj2 (group* or level* or classification* or high* or middle or low*)).tw,kw. |  |  |  |  |  |
| 203 | (educational adj2 status*).tw,kw. |  |  |  |  |  |
| 204 | literac*.tw,kw. |  |  |  |  |  |
| 205 | (consumer price adj1 (index* or indices)).tw,kw. |  |  |  |  |  |
| 206 | (cost* adj2 living).tw,kw. |  |  |  |  |  |
| 207 | household consumption*.tw,kw. |  |  |  |  |  |
| 208 | (utility adj1 theor*).tw,kw. |  |  |  |  |  |
| 209 | compensation*.tw,kw. |  |  |  |  |  |
| 210 | (resource adj2 allocation*).tw,kw. |  |  |  |  |  |
| 211 | (allocative adj1 efficiency).tw,kw. |  |  |  |  |  |
| 212 | ((healthcare or health care) adj1 ration*).tw,kw. |  |  |  |  |  |
| 213 | expropriation*.tw,kw. |  |  |  |  |  |
| 214 | (marginal adj1 analys?s).tw,kw. |  |  |  |  |  |
| 215 | (cost* adj2 (analys?s or benefit*)).tw,kw. |  |  |  |  |  |
| 216 | (cost* adj1 (effectiveness or comparison* or measure* or allocation* or apportionment* or shifting* or containment* or control* or sharing or saving*)).tw,kw. |  |  |  |  |  |
| 217 | (out of pocket adj1 (cost* or pay* or spending)).tw,kw. |  |  |  |  |  |
| 218 | (expenditure* or expense*).tw,kw. |  |  |  |  |  |
| 219 | (return on investment or roi).tw,kw. |  |  |  |  |  |
| 220 | affordabilit*.tw,kw. |  |  |  |  |  |
| 221 | pricing.tw,kw. |  |  |  |  |  |
| 222 | ((medical or health* or treatment or drug or hospital) adj2 cost*).tw,kw. |  |  |  |  |  |
| 223 | (direct service adj1 cost*).tw,kw. |  |  |  |  |  |
| 224 | (charge* or fee or fees).tw,kw. |  |  |  |  |  |
| 225 | (rate set* adj2 review).tw,kw. |  |  |  |  |  |
| 226 | ((group or shared or joint) adj1 purchasing).tw,kw. |  |  |  |  |  |
| 227 | (health adj2 marketing).tw,kw. |  |  |  |  |  |
| 228 | ((healthcare or health care) adj1 (industr* or market* or sector*)).tw,kw. |  |  |  |  |  |
| 229 | investment*.tw,kw. |  |  |  |  |  |
| 230 | (federal adj2 aid*).tw,kw. |  |  |  |  |  |
| 231 | social welfare.tw,kw. |  |  |  |  |  |
| 232 | (social adj1 (security or insurance)).tw,kw. |  |  |  |  |  |
| 233 | (aid adj3 families with dependent children).tw,kw. |  |  |  |  |  |
| 234 | (family adj1 allowance*).tw,kw. |  |  |  |  |  |
| 235 | ((psychological or psychosocial or social) adj1 support*).tw,kw. |  |  |  |  |  |
| 236 | (coping adj2 (behavio?r* or abilit* or strateg*)).tw,kw. |  |  |  |  |  |
| 237 | ((behavio?r* or abilit* or competenc* or skill*) adj1 social).tw,kw. |  |  |  |  |  |
| 238 | ((psychological or emotional or personal) adj adjustment*).tw,kw. |  |  |  |  |  |
| 239 | (emotional adj (adaptation or equilibrium)).tw,kw. |  |  |  |  |  |
| 240 | (behavio?r adj1 (permissive or development or facilitation or pattern or variable)).tw,kw. |  |  |  |  |  |
| 241 | (behavio?r* adj (variable or activity or characteristic or response or specificity or symptoms)).tw,kw. |  |  |  |  |  |
| 242 | (attitude adj2 (health or illness or sickness or patient* or dental or dentist*)).tw,kw. |  |  |  |  |  |
| 243 | (psychological adj3 (phenomena or well* or well-being)).tw,kw. |  |  |  |  |  |
| 244 | (interpersonal adj1 (skill* or relation* or communication*)).tw,kw. |  |  |  |  |  |
| 245 | (rejection* or permissiveness).tw,kw. |  |  |  |  |  |
| 246 | leisure*.tw,kw. |  |  |  |  |  |
| 247 | (festival* or holiday*).tw,kw. |  |  |  |  |  |
| 248 | recreation*.tw,kw. |  |  |  |  |  |
| 249 | relaxation*.tw,kw. |  |  |  |  |  |
| 250 | (hobby or hobbies).tw,kw. |  |  |  |  |  |
| 251 | (sport* or athletic*).tw,kw. |  |  |  |  |  |
| 252 | efficiency.tw,kw. |  |  |  |  |  |
| 253 | productivity.tw,kw. |  |  |  |  |  |
| 254 | ((accident* or emergenc*) adj2 service*).tw,kw. |  |  |  |  |  |
| 255 | (emergency adj2 care).tw,kw. |  |  |  |  |  |
| 256 | emergicenter*.tw,kw. |  |  |  |  |  |
| 257 | advanced trauma life support.tw,kw. |  |  |  |  |  |
| 258 | (emergency adj2 dispatch*).tw,kw. |  |  |  |  |  |
| 259 | (ems adj1 communication adj1 system*).tw,kw. |  |  |  |  |  |
| 260 | (emergency adj1 hospital adj1 service*).tw,kw. |  |  |  |  |  |
| 261 | (emergency adj1 (ward* or department* or room* or centre* or center* or dispensar*)).tw,kw. |  |  |  |  |  |
| 262 | (trauma adj1 (center* or centre*)).tw,kw. |  |  |  |  |  |
| 263 | (emergency adj2 unit*).tw,kw. |  |  |  |  |  |
| 264 | (emergency adj3 (visit* or admission*)).tw,kw. |  |  |  |  |  |
| 265 | (emergency adj1 psychiatric adj1 service*).tw,kw. |  |  |  |  |  |
| 266 | (patient* adj2 transport*).tw,kw. |  |  |  |  |  |
| 267 | (wounded adj2 sick adj2 transport*).tw,kw. |  |  |  |  |  |
| 268 | triage*.tw,kw. |  |  |  |  |  |
| 269 | (organizational adj1 involvement*).tw,kw. |  |  |  |  |  |
| 270 | (health adj3 indicator*).tw,kw. |  |  |  |  |  |
| 271 | (time adj4 loss).tw,kw. |  |  |  |  |  |
| 272 | or/128-270 |  |  |  |  |  |
| 273 | 126 and 272 |  |  |  |  |  |
| 274 | limit 273 to animals |  |  |  |  |  |
| 275 | 273 not 274 |  |  |  |  |  |
| 276 | limit 275 to (english language and yr="1999 -Current" and (article or article in press)) |  |  |  |  |  |
| 277 | limit 276 to (evidence based medicine or consensus development or meta analysis or outcomes research or "systematic review" or "qualitative (best balance of sensitivity and specificity)") |  |  |  |  |  |
| 278 | Clinical study/ or Case control study/ or Family study/ or Longitudinal study/ or Retrospective study/ or (Prospective study/ not Randomized controlled trials/) or Cohort analysis/ or (Cohort adj (study or studies)).mp. or (Case control adj (study or studies)).tw. or (follow up adj (study or studies)).tw. or (observational adj (study or studies)).tw. or (epidemiologic$ adj (study or studies)).tw. or (cross sectional adj (study or studies)).tw. |  |  |  |  |  |
| 279 | 276 and 278 |  |  |  |  |  |
| 280 | 277 or 279 |  |  |  |  |  |
| 281 | (Equipment* or device* or supply or supplies or t??th brush* or t??thbrush or tooth paste or toothpaste or versus).ti. |  |  |  |  |  |
| 282 | (rats or rat or mice or dog* or pig* or horse* or dog* or mouse or rabbit* or animal*).tw,kw. |  |  |  |  |  |
| 283 | (dental adj2 (student* or school* or university or universities or college*)).tw,kw. |  |  |  |  |  |
| 284 | 281 or 282 or 283 |  |  |  |  |  |
| 285 | 280 not 284 |  |  |  |  |  |

**Key**

/ = indexing term (Emtree heading)

exp = exploded indexing term (Emtree heading)

* = truncation

? = wildcard symbol as a substitute for one character or none

tw = text word search in title or abstract fields

kw =  keywords defined by the author of the article

pt = publication type.

adjn = terms within (n-1) words of each other (any order)

**CINAHL:**

**Language:** English

**Age group:** No limitation

**Publication year:** after 1999

**Publication Types:** Evidence-Based Care Sheet**,** Meta-analysis, Meta Synthesis, Systematic Reviews

**Clinical Queries**: Review (best balance of sensitivity and specificity), Qualitative: (best balance of sensitivity and specificity)

**External filters used:** qualitative studies, cohort studies, case control studies

**Exclusion**: equipment and supplies, all animal studies, dental students, dental schools

**Searched on Aug.12 2019, Jan. 20, 2020, Feb. 18, 2022**

**Records retrieved:** 5370 (Aug. 2019); 73 (Jan. 2020); 506 (Feb. 2022)

**Interface - EBSCOhost Research Databases 
Database - CINAHL Plus with Full Text**Search history sorted by search number ascending

| # | Searches | Limiters |
| --- | --- | --- |
| 1 | (MH "Dental Care+") |  |
| 2 | (MH "Dental Clinics") |  |
| 3 | (MH "Public Health Dentistry") OR (MH "Dental Health Education") |  |
| 4 | (MH "Diagnosis, Oral+") |  |
| 5 | (MH "Dental Prophylaxis+") |  |
| 6 | (MH "Dental Health Services+") |  |
| 7 | (MH "Dentistry, Operative+") |  |
| 8 | (MH "Endodontics+") |  |
| 9 | (MH "Esthetics, Dental") |  |
| 10 | (MH "Surgery, Oral+") |  |
| 11 | (MH "Orthodontics+") |  |
| 12 | (MH "Pathology, Oral") |  |
| 13 | (MH "Periodontics+") |  |
| 14 | (MH "Preventive Dentistry+") |  |
| 15 | (MH "Prosthodontics+") |  |
| 16 | (MH "Oral Medicine") |  |
| 17 | 1 OR 2 OR 3 OR 4 OR 5 OR 6 OR 7 OR 8 OR 9 OR 10 OR 11 OR 12 OR 13 OR 14 OR 15 OR 16 |  |
| 18 | (MH "Health Promotion+") |  |
| 19 | (MH "Public Health") |  |
| 20 | (MH "Patient Education+") |  |
| 21 | (MH "Dentistry+") |  |
| 22 | 18 OR 19 OR 20 |  |
| 23 | 21 AND 22 |  |
| 24 | 22 | **Limiters** - Special Interest: Dental Care |
| 25 | 23 OR 24 |  |
| 26 | 17 OR 25 |  |
| 27 | TI care N2 dental OR AB care N2 dental |  |
| 28 | TI dental N2 service* OR AB dental N2 service* |  |
| 29 | TI ( dent* N1 (“check-up” or health or intervention* or clinic* or treatment* or therap* or program* or practice* or education or procedure* or restorat* or regenerative*) ) OR AB ( dent* N1 (“check-up” or health or intervention* or clinic* or treatment* or therap* or program* or practice* or education or procedure* or restorat* or regenerative*) ) |  |
| 30 | TI dental stress analys#s OR AB dental stress analys#s |  |
| 31 | TI denture* OR AB denture* |  |
| 32 | TI oral N1 care OR AB oral N1 care |  |
| 33 | TI “dental health” N1 survey* OR AB “dental health” N1 survey* |  |
| 34 | TI ( (decayed or missing or filled) N2 teeth ) OR AB ( (decayed or missing or filled) N2 teeth ) |  |
| 35 | TI “bleeding on probing” N2 gingival OR AB “bleeding on probing” N2 gingival |  |
| 36 | TI ( (periodont* or orthodontic* or prosthodont* or endodontic) N1 (treatment* or procedure* or surger* or care) ) OR AB ( (periodont* or orthodontic* or prosthodont* or endodontic) N1 (treatment* or procedure* or surger* or care) ) |  |
| 37 | TI ( (dental or t??th or periodontal) N0 cleaning ) OR AB ( (dental or t??th or periodontal) N0 cleaning ) |  |
| 38 | TI ( endodontics or orthodontics or periodontics or prosthodontics ) OR AB ( endodontics or orthodontics or periodontics or prosthodontics ) |  |
| 39 | TI ( orthodontia or orthodontolog* or orthodonty ) OR AB ( orthodontia or orthodontolog* or orthodonty ) |  |
| 40 | TI ( Orthodontic N1 (“space closure” or anchorage* or extrusion*) ) OR AB ( Orthodontic N1 (“space closure” or anchorage* or extrusion*) ) |  |
| 41 | TI ( root canal N0 (therap* or procedure* or obturation* or preparation* or surger*) ) OR AB ( root canal N0 (therap* or procedure* or obturation* or preparation* or surger*) ) |  |
| 42 | TI ( (oral or mouth) N1 surg* ) OR AB ( (oral or mouth) N1 surg* ) |  |
| 43 | TI parotidectom* OR AB parotidectom* |  |
| 44 | TI ( (“pre prosthetic” or preprosthetic) N0 surger* ) OR AB ( (“pre prosthetic” or preprosthetic) N0 surger* ) |  |
| 45 | TI “cavity lining*” N0 dental OR AB “cavity lining*” N0 dental |  |
| 46 | TI varnish* N1 cavity OR AB varnish* N1 cavity |  |
| 47 | TI ( “community periodontal index of treatment needs” or cpitn ) OR AB ( “community periodontal index of treatment needs” or cpitn ) |  |
| 48 | TI ( (mouth or oral) N1 rehabilitation* ) OR AB ( (mouth or oral) N1 rehabilitation* ) |  |
| 49 | TI ( (diagnos* or examination*) N0 (oral or dental) ) OR AB ( (diagnos* or examination*) N0 (oral or dental) ) |  |
| 50 | TI dental N0 pulp test* OR AB dental N0 pulp test* |  |
| 51 | TI ( (dental or intraoral or orthodontic) N0 photograph* ) OR AB ( (dental or intraoral or orthodontic) N0 photograph* ) |  |
| 52 | TI ( (dental or bitewing or tooth) N0 (radiography or radiovisiography) ) OR AB ( (dental or bitewing or tooth) N0 (radiography or radiovisiography) ) |  |
| 53 | TI ( Dental N0 (“x ray” or xray) ) OR AB ( Dental N0 (“x ray” or xray) ) |  |
| 54 | TI fluorescence N0 “quantitative light-induced” OR AB fluorescence N0 “quantitative light-induced” |  |
| 55 | TI community N0 dentistry OR AB community N0 dentistry |  |
| 56 | TI ( (dental or periodontal or t??th) N0 prophylaxis ) OR AB ( (dental or periodontal or t??th) N0 prophylaxis ) |  |
| 57 | TI ( (supragingival or subgingival or dental or root) N0 scaling* ) OR AB ( (supragingival or subgingival or dental or root) N0 scaling* ) |  |
| 58 | TI ( (maxillo-mandibular or maxillomandibular or oral or maxillofacial or orthognathic or jaw) N1 surg* ) OR AB ( (maxillo-mandibular or maxillomandibular or oral or maxillofacial or orthognathic or jaw) N1 surg* ) |  |
| 59 | TI ( (maxillo-mandibular or maxillomandibular or oral or maxillofacial or orthognathic) N1 procedure* ) OR AB ( (maxillo-mandibular or maxillomandibular or oral or maxillofacial or orthognathic) N1 procedure* ) |  |
| 60 | TI oral N0 hygiene OR AB oral N0 hygiene |  |
| 61 | TI ( (evidence-based or preventative or cosmetic or operative or prosthetic or reparative) N1 dentistry ) OR AB ( (evidence-based or preventative or cosmetic or operative or prosthetic or reparative) N1 dentistry ) |  |
| 62 | TI ( dental N0 (esthetic* or aesthetic* or floss or device* or scaling* or brace* or hygiene* or polishing or radiology or bonding or soldering* or prosthetic*) ) OR AB ( dental N0 (esthetic* or aesthetic* or floss or device* or scaling* or brace* or hygiene* or polishing or radiology or bonding or soldering* or prosthetic*) ) |  |
| 63 | TI ( (dental or tooth) N2 filling* ) OR AB ( (dental or tooth) N2 filling* ) |  |
| 64 | TI ( (dental or tooth or caries) N1 prevention ) OR AB ( (dental or tooth or caries) N1 prevention ) |  |
| 65 | TI ( (tooth or dental) N0 (an#esthesia or an#esthetic* or casting*) ) OR AB ( (tooth or dental) N0 (an#esthesia or an#esthetic* or casting*) ) |  |
| 66 | TI “Jaw relation record*” OR AB “Jaw relation record*” |  |
| 67 | TI ( Sinus N0 (“floor augmentation*” or lifting*) ) OR AB ( Sinus N0 (“floor augmentation*” or lifting*) ) |  |
| 68 | TI ( dental N1 (implant* or prosthes#s) ) OR AB ( dental N1 (implant* or prosthes#s) ) |  |
| 69 | TI ( (dental or enamel) N0 microabrasion* ) OR AB ( (dental or enamel) N0 microabrasion* ) |  |
| 70 | TI ( T??th N0 (bleaching or whitening or replantation* or reimplantation* or extrusion* or uprighting* or remineralization* or polishing or brushing or restoration* or surger* or inlay* or preparation* or removal* or resection*)) ) OR AB ( T??th N0 (bleaching or whitening or replantation* or reimplantation* or extrusion* or uprighting* or remineralization* or polishing or brushing or restoration* or surger* or inlay* or preparation* or removal* or resection*)) ) |  |
| 71 | TI ( Dental N0 (reimplantation* or replantation* or reinclusion*) ) OR AB ( Dental N0 (reimplantation* or replantation* or reinclusion*) ) |  |
| 72 | TI dental N0 infection N0 control* OR AB dental N0 infection N0 control* |  |
| 73 | TI ( apicoectom* or gingivectom* or gingivoplast* or glossectom* ) OR AB ( apicoectom* or gingivectom* or gingivoplast* or glossectom* ) |  |
| 74 | TI ( (tongue or lingual) N0 (extirpation* or resection*) ) OR AB ( (tongue or lingual) N0 (extirpation* or resection*) ) |  |
| 75 | TI ( (jaw or maxillomandibular) N0 fixation* ) OR AB ( (jaw or maxillomandibular) N0 fixation* ) |  |
| 76 | TI ( mandibula* N0 (advancement* or reconstruction* or restoration* or resection*) ) OR AB ( mandibula* N0 (advancement* or reconstruction* or restoration* or resection*) ) |  |
| 77 | TI ( “mandible ostectom*” or mandibulectom* ) OR AB ( “mandible ostectom*” or mandibulectom* ) |  |
| 78 | TI ( (maxillofacial or mandibular) N0 (prosthes#s or impant*) ) OR AB ( (maxillofacial or mandibular) N0 (prosthes#s or impant*) ) |  |
| 79 | TI ( (upper jaw or maxilla*) N0 resection) ) OR AB ( (upper jaw or maxilla*) N0 resection) ) |  |
| 80 | TI maxillectom* OR AB maxillectom* |  |
| 81 | TI genioplast* OR AB genioplast* |  |
| 82 | TI ( chin N1 (correction* or reconstruction * or reduction* or surger* or operation*) ) OR AB ( chin N1 (correction* or reconstruction * or reduction* or surger* or operation*) ) |  |
| 83 | TI ( chinplast* or mentoplast* ) OR AB ( chinplast* or mentoplast* ) |  |
| 84 | TI ( (maxillary or mandibular or alveolar) N0 ridge augmentation* ) OR AB ( (maxillary or mandibular or alveolar) N0 ridge augmentation* ) |  |
| 85 | TI ( alveolectom* or alveoloplast* or vestibuloplast* ) OR AB ( alveolectom* or alveoloplast* or vestibuloplast* ) |  |
| 86 | TI ( (endosseous or subperiosteal) N1 implant* ) OR AB ( (endosseous or subperiosteal) N1 implant* ) |  |
| 87 | TI alveolar N1 graft* OR AB alveolar N1 graft* |  |
| 88 | TI ( (“le fort” or lefort or maxilla* or jaw or mandib*) N1 osteotom* ) OR AB ( (“le fort” or lefort or maxilla* or jaw or mandib*) N1 osteotom* ) |  |
| 89 | TI “Le fort” N0 operation* OR AB “Le fort” N0 operation* |  |
| 90 | TI “sagittal split” N1 osteotom* OR AB “sagittal split” N1 osteotom* |  |
| 91 | TI sinus N1 augmentation* OR AB sinus N1 augmentation* |  |
| 92 | TI ( (t??th or serial) N0 extraction* ) OR AB ( (t??th or serial) N0 extraction* ) |  |
| 93 | TI Exodont* OR AB Exodont* |  |
| 94 | TI ( Molar N0 (amputation* or extraction*) ) OR AB ( Molar N0 (amputation* or extraction*) ) |  |
| 95 | TI odontectom* OR AB odontectom* |  |
| 96 | TI ( uvulopharyngopalatoplast* or uvulopalatoplast* or palatouvulopharyngoplast* or pharyngouvulopalatoplast* ) OR AB ( uvulopharyngopalatoplast* or uvulopalatoplast* or palatouvulopharyngoplast* or pharyngouvulopalatoplast* ) |  |
| 97 | TI ( dental N0 (internal or marginal) N0 adaptation* ) OR AB ( dental N0 (internal or marginal) N0 adaptation* ) |  |
| 98 | TI mandibular N0 advancement OR AB mandibular N0 advancement |  |
| 99 | TI ( occlusal N0 (splint* or adjustment* or equilibration*) ) OR AB ( occlusal N0 (splint* or adjustment* or equilibration*) ) |  |
| 100 | TI ( (fixed or activator or extraoral) N1 appliance* ) OR AB ( (fixed or activator or extraoral) N1 appliance* ) |  |
| 101 | TI ( (fixed or bonded or permanent) N0 retainer* ) OR AB ( (fixed or bonded or permanent) N0 retainer* ) |  |
| 102 | TI ( (clear aligner or herbst or bimler or andresen) N0 appliance* ) OR AB ( (clear aligner or herbst or bimler or andresen) N0 appliance* ) |  |
| 103 | TI frankel N1 regulator* OR AB frankel N1 regulator* |  |
| 104 | TI ( (function or harvold) N0 activator* ) OR AB ( (function or harvold) N0 activator* ) |  |
| 105 | TI jasper N0 jumper* OR AB jasper N0 jumper* |  |
| 106 | TI bionator* OR AB bionator* |  |
| 107 | TI invisalign* OR AB invisalign* |  |
| 108 | TI crown N0 lengthening OR AB crown N0 lengthening |  |
| 109 | TI forced N1 eruption* OR AB forced N1 eruption* |  |
| 110 | TI ( (maxillary or palatal) N0 expansion* ) OR AB ( (maxillary or palatal) N0 expansion* ) |  |
| 111 | TI palatoplast* OR AB palatoplast* |  |
| 112 | TI ( palate N0 (plast* or reconstruction* or operation*) ) OR AB ( palate N0 (plast* or reconstruction* or operation*) ) |  |
| 113 | TI ( tooth N1 (movement* or intrusion* or depression* or care) ) OR AB ( tooth N1 (movement* or intrusion* or depression* or care) ) |  |
| 114 | TI ( (oral or maxillofacial) N2 patholog* ) OR AB ( (oral or maxillofacial) N2 patholog* ) |  |
| 115 | TI ( periodontal N0 (medicine*or prosthes#s or splint* or dressing* or prevention*) ) OR AB ( periodontal N0 (medicine*or prosthes#s or splint* or dressing* or prevention*) ) |  |
| 116 | TI ( periodontal N1 (“tissue regeneration*” or debridement*) ) OR AB ( periodontal N1 (“tissue regeneration*” or debridement*) ) |  |
| 117 | TI ( (dental or mouth or t??th) N0 debridement* ) OR AB ( (dental or mouth or t??th) N0 debridement* ) |  |
| 118 | TI root N1 planing* OR AB root N1 planing* |  |
| 119 | TI ( (subgingival or gingival) N0 (curettage* or retraction*) ) OR AB ( (subgingival or gingival) N0 (curettage* or retraction*) ) |  |
| 120 | TI fluoridation* OR AB fluoridation* |  |
| 121 | TI ( mouth N0 (protect* or guard) ) OR AB ( mouth N0 (protect* or guard) ) |  |
| 122 | TI toothbrushing* OR AB toothbrushing* |  |
| 123 | TI stomatolog* OR AB stomatolog* |  |
| 124 | TI medicine N0 oral OR AB medicine N0 oral |  |
| 125 | 27 OR 28 OR 29 OR 30 OR 31 OR 32 OR 33 OR 34 OR 35 OR 36 OR 37 OR 38 OR 39 OR 40 OR 41 OR 42 OR 43 OR 44 OR 45 OR 46 OR 47 OR 48 OR 49 OR 50 OR 51 OR 52 OR 53 OR 54 OR 55 OR 56 OR 57 OR 58 OR 59 OR 60 OR 61 OR 62 OR 63 OR 64 OR 65 OR 66 OR 67 OR 68 OR 69 OR 70 OR 71 OR 72 OR 73 OR 74 OR 75 OR 76 OR 77 OR 78 OR 79 OR 80 OR 81 OR 82 OR 83 OR 84 OR 85 OR 86 OR 87 OR 88 OR 89 OR 90 OR 91 OR 92 OR 93 OR 94 OR 95 OR 96 OR 97 OR 98 [...](javascript:showHistoryTerm('ctl00_ctl00_MainContentArea_MainContentArea_historyControl_HistoryRepeater_ctl162_ellipsis',true)) |  |
| 126 | TI ( (promotion* or campaign* or program*) N1 health ) OR AB ( (promotion* or campaign* or program*) N1 health ) |  |
| 127 | TI program* N1 wellness OR AB program* N1 wellness |  |
| 128 | TI “head start” N0 program* OR AB “head start” N0 program* |  |
| 129 | TI ( Pathology or pathological or telepathology ) OR AB ( Pathology or pathological or telepathology ) |  |
| 130 | TI ( (public or deliver* or system*) N0 (“health care” or healthcare) ) OR AB ( (public or deliver* or system*) N0 (“health care” or healthcare) ) |  |
| 131 | 126 OR 127 OR 128 OR 129 OR 130 |  |
| 132 | (MH "Dentistry") |  |
| 133 | 131 AND 132 |  |
| 134 | 131 | **Limiters** - Special Interest: Dental Care |
| 135 | 133 OR 134 |  |
| 136 | 125 OR 135 |  |
| 137 | 26 OR 136 |  |
| 138 | (MH "Drug Utilization+") OR (MH "Utilization Review") |  |
| 139 | (MH "Health Services") |  |
| 140 | (MH "Community Health Services+") |  |
| 141 | (MH "Child Health Services+") |  |
| 142 | (MH "Hospices") |  |
| 143 | (MH "Hospitalization+") OR (MH "Length of Stay") OR (MH "Patient Admission") OR (MH "Patient Discharge") OR (MH "Patient Dumping") OR (MH "Readmission") |  |
| 144 | (MH "Emergency Medical Services+") |  |
| 145 | (MH "Advanced Trauma Life Support Care") |  |
| 146 | (MH "Poison Control Centers") |  |
| 147 | (MH "Rehabilitation") |  |
| 148 | (MH "Activities of Daily Living") OR (MH "Social Participation") |  |
| 149 | (MH "Self Care") OR (MH "Self Care Agency") OR (MH "Self-Management") |  |
| 150 | (MH "Role") OR (MH "Professional Role") OR (MH "Nursing Role") OR (MH "Physician's Role") OR (MH "Sick Role") |  |
| 151 | (MH "Job Satisfaction") |  |
| 152 | (MH "Employment+") OR (MH "Unemployment") |  |
| 153 | (MH "Career Mobility+") |  |
| 154 | (MH "Job Re-Entry") |  |
| 155 | (MH "Downsizing, Organizational") |  |
| 156 | (MH "Work Environment") |  |
| 157 | (MH "Economics+") |  |
| 158 | (MH "Support, Psychosocial") |  |
| 159 | (MH "Leisure Activities+") |  |
| 160 | (MH "Productivity") |  |
| 161 | TI "return on investment" OR AB "return on investment" OR TI ROI OR AB ROI |  |
| 162 | TI affordabilit* OR AB affordabilit* |  |
| 163 | TI pricing OR AB pricing |  |
| 164 | TI ( medical or health* or treatment or drug or hospital) N1 cost* ) OR AB ( medical or health* or treatment or drug or hospital) N1 cost* ) |  |
| 165 | TI debt* OR AB debt* |  |
| 166 | TI ( charge* or fee or fees ) OR AB ( charge* or fee or fees ) |  |
| 167 | TI "direct service" N0 cost* OR AB "direct service" N0 cost* |  |
| 168 | TI ( (group or shared or joint) N0 purchasing ) OR AB ( (group or shared or joint) N0 purchasing ) |  |
| 169 | TI health N1 marketing OR AB health N1 marketing |  |
| 170 | TI ( (healthcare or “health care”) N0 (industr* or market* or sector*) ) OR AB ( (healthcare or “health care”) N0 (industr* or market* or sector*) ) |  |
| 171 | TI investment* OR AB investment* |  |
| 172 | TI federal N1 aid* OR AB federal N1 aid* |  |
| 173 | TI “social welfare” OR AB “social welfare” |  |
| 174 | TI ( social N0 (security or insurance) ) OR AB ( social N0 (security or insurance) ) |  |
| 175 | TI aid N2 “families with dependent children” OR AB aid N2 “families with dependent children” |  |
| 176 | TI family N0 allowance* OR AB family N0 allowance* |  |
| 177 | TI ( (psychological or psychosocial or social) N0 support* ) OR AB ( (psychological or psychosocial or social) N0 support* ) |  |
| 178 | TI leisure* OR AB leisure* |  |
| 179 | TI ( festival* or holiday* ) OR AB ( festival* or holiday* ) |  |
| 180 | TI recreation* OR AB recreation* |  |
| 181 | TI relaxation* OR AB relaxation* |  |
| 182 | TI ( hobby or hobbies ) OR AB ( hobby or hobbies ) |  |
| 183 | TI ( sport* or athletic* ) OR AB ( sport* or athletic* ) |  |
| 184 | TI efficiency OR AB efficiency |  |
| 185 | TI productivity OR AB productivity |  |
| 186 | TI ( (accident* or emergenc*) N1 service* ) OR AB ( (accident* or emergenc*) N1 service* ) |  |
| 187 | TI emergency N1 care OR AB emergency N1 care |  |
| 188 | TI emergicenter* OR AB emergicenter* |  |
| 189 | TI “advanced trauma life support” OR AB “advanced trauma life support” |  |
| 190 | TI emergency N1 dispatch* OR AB emergency N1 dispatch* |  |
| 191 | TI emergency N0 hospital N0 service* OR AB emergency N0 hospital N0 service* |  |
| 192 | TI ( emergency N0 (ward* or department* or room* or centre* or center* or dispensar*) ) OR AB ( emergency N0 (ward* or department* or room* or centre* or center* or dispensar*) ) |  |
| 193 | TI ( trauma N0 (center* or centre*) ) OR AB ( trauma N0 (center* or centre*) ) |  |
| 194 | TI emergency N1 unit OR AB emergency N1 unit |  |
| 195 | TI ( emergency N2 (visit* or admission*) ) OR AB ( emergency N2 (visit* or admission*) ) |  |
| 196 | TI emergency N0 psychiatric N1 service* OR AB emergency N0 psychiatric N1 service* |  |
| 197 | TI patient* N1 transport* OR AB patient* N1 transport* |  |
| 198 | TI triage* OR AB triage* |  |
| 199 | TI organizational N0 involvement* OR AB organizational N0 involvement* |  |
| 200 | TI health N2 indicator*lth N2 indicator* OR AB health N2 indicator* |  |
| 201 | TI time N3 loss OR AB time N3 loss |  |
| 202 | TI responsiveness OR AB responsiveness |  |
| 203 | TI drug N2 utilization OR AB drug N2 utilization |  |
| 204 | TI ( (drug or medicine) N1 (us* or misus* or consumption*) ) OR AB ( (drug or medicine) N1 (us* or misus* or consumption*) ) |  |
| 205 | TI health N2 system* OR AB health N2 system* |  |
| 206 | TI ( health N1 (service* or agenc* or practice*) ) OR AB ( health N1 (service* or agenc* or practice*) ) |  |
| 207 | TI ( (preventative or physician* or diagnostic) N1 service* ) OR AB ( (preventative or physician* or diagnostic) N1 service* ) |  |
| 208 | TI medical N1 overus* OR AB medical N1 overus* |  |
| 209 | TI ( rehabilitation* or readaptation* or readjustment* or recover* or resociali#ation* or revalidation* ) OR AB ( rehabilitation* or readaptation* or readjustment* or recover* or resociali#ation* or revalidation* ) |  |
| 210 | TI ( functional N0 (assessment* or training*) ) OR AB ( functional N0 (assessment* or training*) ) |  |
| 211 | TI ( community N1 (health or care) ) OR AB ( community N1 (health or care) ) |  |
| 212 | TI ( community N0 (integration* or reintegration* or program*) ) OR AB ( community N0 (integration* or reintegration* or program*) ) |  |
| 213 | TI ( daycare N0 (center* or centre*) ) OR AB ( daycare N0 (center* or centre*) ) |  |
| 214 | TI ( (“public health” or community) N1 nursing ) OR AB ( (“public health” or community) N1 nursing ) |  |
| 215 | TI assertive N0 community N0 treatment* OR AB assertive N0 community N0 treatment* |  |
| 216 | TI community N0 network* OR AB community N0 network* |  |
| 217 | TI community N0 pharmac* N0 service* OR AB community N0 pharmac* N0 service* |  |
| 218 | TI family N0 planning OR AB family N0 planning |  |
| 219 | TI ( planned N0 (pregnanc* or parenthood) ) OR AB ( planned N0 (pregnanc* or parenthood) ) |  |
| 220 | TI ( (foster or kinship) N1 care ) OR AB ( (foster or kinship) N1 care ) |  |
| 221 | TI ( (home or domiciliary) N0 care ) OR AB ( (home or domiciliary) N0 care ) |  |
| 222 | TI Hospice* OR AB Hospice* |  |
| 223 | TI Maternal N0 health N0 service* OR AB Maternal N0 health N0 service* |  |
| 224 | TI ( (employment or occupational or employee*) N2 (health or assistance) ) OR AB ( (employment or occupational or employee*) N2 (health or assistance) ) |  |
| 225 | TI ( (emergency or evacuation) N0 shelter* ) OR AB ( (emergency or evacuation) N0 shelter* ) |  |
| 226 | TI ( (“displaced person*” or refugee) N0 (camp* or settlement* or shelter*) ) OR AB ( (“displaced person*” or refugee) N0 (camp* or settlement* or shelter*) ) |  |
| 227 | TI ( (senior or aged or elderly) N2 (centre* or center* or home* or facilit*) ) OR AB ( (senior or aged or elderly) N2 (centre* or center* or home* or facilit*) ) |  |
| 228 | TI “Uncompensated care” OR AB “Uncompensated care” |  |
| 229 | TI ( (work or job or vocation* or life) N2 (satisfaction* or performance* or stress*) ) OR AB ( (work or job or vocation* or life) N2 (satisfaction* or performance* or stress*) ) |  |
| 230 | TI employment OR AB employment |  |
| 231 | TI labo#r force OR AB labo#r force |  |
| 232 | TI occupational N1 status OR AU occupational N1 status |  |
| 233 | TI underemployment OR AB underemployment |  |
| 234 | TI ( (career or clinical or job) N0 ladder* ) OR AB ( (career or clinical or job) N0 ladder* ) |  |
| 235 | TI career N0 mobilit* OR AB career N0 mobilit* |  |
| 236 | TI ( (back or return) N1 work ) OR AB ( (back or return) N1 work ) |  |
| 237 | TI ( health N0 (resource* or workforce) ) OR AB ( health N0 (resource* or workforce) ) |  |
| 238 | TI health N1 manpower OR AB health N1 manpower |  |
| 239 | TI hospitali#ation* OR AB hospitali#ation* |  |
| 240 | TI hospital N1 service* OR AB hospital N1 service* |  |
| 241 | TI hospital stay* OR AB hospital stay* |  |
| 242 | TI length* N1 stay* OR AB length* N1 stay* |  |
| 243 | TI ( (patient or voluntary) N0 admission* ) OR AB ( (patient or voluntary) N0 admission* ) |  |
| 244 | TI ( (hospital or patient) N0 readmission* ) OR AB ( (hospital or patient) N0 readmission* ) |  |
| 245 | TI economic* OR AB economic* |  |
| 246 | TI ( (macroeconomic or microeconomic or socioeconomic) N0 (factor* or aspect*) ) OR AB ( (macroeconomic or microeconomic or socioeconomic) N0 (factor* or aspect*) ) |  |
| 247 | TI ( income N1 (group* or level* or classification* or high* or middle or low*) ) OR AB ( income N1 (group* or level* or classification* or high* or middle or low*) ) |  |
| 248 | TI ( (high* or middle or low* or develop*) N1 countr* ) OR AB ( (high* or middle or low* or develop*) N1 countr* ) |  |
| 249 | TI educational N1 status* OR AB educational N1 status* |  |
| 250 | TI literac* OR AB literac* |  |
| 251 | TI ( “consumer price” N0 (index* or indices) ) OR AB ( “consumer price” N0 (index* or indices) ) |  |
| 252 | TI Consumption OR AB Consumption |  |
| 253 | TI cost* N1 living OR AB cost* N1 living |  |
| 254 | TI easterlin N0 hypothesis OR AB easterlin N0 hypothesis |  |
| 255 | TI “household consumption*” OR AB “household consumption*” |  |
| 256 | TI productivity OR AB productivity |  |
| 257 | TI utility N0 theor* OR AB utility N0 theor* |  |
| 258 | TI compensation* OR AB compensation* |  |
| 259 | TI resource N1 allocation* OR AB resource N1 allocation* |  |
| 260 | TI allocative N0 efficiency OR AB allocative N0 efficiency |  |
| 261 | TI ( (healthcare or “health care”) N0 ration* ) OR AB ( (healthcare or “health care”) N0 ration* ) |  |
| 262 | TI expropriation* OR AB expropriation* |  |
| 263 | TI marginal N0 analys#s OR AB marginal N0 analys#s |  |
| 264 | TI ( cost* N1 (analys#s or benefit*) ) OR AB ( cost* N1 (analys#s or benefit*) ) |  |
| 265 | TI ( cost* N0 (effectiveness or comparison* or measure* or allocation* or apportionment* or shifting* or containment* or control* or sharing or saving*) ) OR AB ( cost* N0 (effectiveness or comparison* or measure* or allocation* or apportionment* or shifting* or containment* or control* or sharing or saving*) ) |  |
| 266 | TI ( “out of pocket” N0 (cost* or pay* or spending) ) OR AB ( “out of pocket” N0 (cost* or pay* or spending) ) |  |
| 267 | TI ( expenditure* or expense* ) OR AB ( expenditure* or expense* ) |  |
| 268 | (MH "Absenteeism") OR (MH "Presenteeism") |  |
| 269 | TI ( absenteeism or presenteeism ) OR AB ( absenteeism or presenteeism ) |  |
| 270 | (MH "Health Status") OR (MH "Health Status Disparities") |  |
| 271 | TI health N0 status OR AB health N0 status |  |
| 272 | 138 OR 139 OR 140 OR 141 OR 142 OR 143 OR 144 OR 145 OR 146 OR 147 OR 148 OR 149 OR 150 OR 151 OR 152 OR 153 OR 154 OR 155 OR 156 OR 157 OR 158 OR 159 OR 160 OR 161 OR 162 OR 163 OR 164 OR 165 OR 166 OR 167 OR 168 OR 169 OR 170 OR 171 OR 172 OR 173 OR 174 OR 175 OR 176 OR 177 OR 178 OR 179 OR 180 OR 181 OR 182 OR 183 OR 184 OR 185 OR 186 OR 187 OR 188 OR 189 OR 190 OR 191 OR 192 OR 193 OR 194 OR 195 OR 196 OR 197 OR 198 OR 199 OR 200 [...](javascript:showHistoryTerm('ctl00_ctl00_MainContentArea_MainContentArea_historyControl_HistoryRepeater_ctl15_ellipsis',true)) |  |
| 273 | 137 AND 272 |  |
| 274 | 273 | **Limiters** - Published Date: 19990101-20190831; English Language |
| 275 | 274 | **Limiters** - Publication Type: Evidence-Based Care Sheet, Meta Analysis, Meta Synthesis, Systematic Review |
| 276 | (MH "Case Control Studies+") or (MH "Control Group") or (MH "Matched-Pair Analysis") or (TI (case or cases) n5 TI (control or controls)) OR (AB (case or cases) n5 AB (control or controls)) OR (TI (case or cases) n3 TI (matched)) OR (AB (case or cases) n3 AB (matched)) OR TI (control group*) |  |
| 277 | (TI (cohort)) OR (AB (cohort)) OR (MH "Cohort Studies") OR (TI (longitudinal)) OR (AB (longitudinal)) OR (TI (prospective)) OR (AB (prospective)) OR (TI (retrospective)) OR (AB (retrospective)) |  |
| 278 | (TI (interview)) OR (AB (interview)) OR (MH "Audiorecording") OR (TI ("qualitative stud$")) OR (AB ("qualitative stud$")) |  |
| 279 | 276 OR 277 OR 278 |  |
| 280 | 274 AND 279 |  |
| 281 | 274 | **Limiters** - Clinical Queries: Review - Best Balance, Qualitative - Best Balance |
| 282 | 275 OR 280 OR 281 |  |
| 283 | TI ( rats or rat or mice or dog* or pig or horse* or mouse or rabbit or animal* ) OR AB ( rats or rat or mice or dog* or pig or horse* or mouse or rabbit or animal*) ) |  |
| 284 | TI ( Equipment* or instrument* or device* or supply or supplies or t##th brush* or t##thbrush or tooth paste or toothpaste or versus ) OR AB ( Equipment* or instrument* or device* or supply or supplies or t##th brush* or t##thbrush or tooth paste or toothpaste or versus ) |  |
| 285 | TI ( dental N1 (student* or school* or university or universities or college*) ) OR AB ( dental N1 (student* or school* or university or universities or college*) ) |  |
| 286 | 283 OR 284 OR 285 |  |
| 287 | 282 NOT 286 |  |

**Key**

MH = indexing term (CINAHL heading)

* = truncation

TI = terms in the title

AB = terms in the abstract

“ “ = phrase search.

Nn = terms within n words of each other (any order)

$ = Mandatory wildcards (stands for exactly 1 character)

? = Mandatory wildcards (stands for exactly 1 character)

# = Optional wildcards (stands for 1 or 0 characters)

**Sociological Abstracts**

**Language: English**

**Publication year: after 1999**

**Searched on Aug.27, 2019, Jan. 21, 2020, Feb 19, 2022**

**Records retrieved:** 372 (Aug. 2019); 3 (Jan. 2020); 55 (Feb. 2022)

**Search Strategy:**

su(dental care) OR ((noft((care NEAR/2 dental) OR (dental NEAR/2 service*) OR (dental NEAR/1 "check up") OR (dental NEAR/1 (health OR intervention* OR clinic* OR treatment* OR therap* OR program* OR practice* OR education OR procedure* OR restorat* OR regenerative*)) OR (dental stress analys?s) OR denture* OR (oral NEAR/1 care) OR ("dental health" NEAR/1 survey*) OR ((decayed OR missing OR filled) NEAR/2 teeth) OR endodontics OR orthodontics OR periodontics OR prosthodontics OR ((periodont* OR orthodontic* OR prosthodont* OR endodontic) NEAR/1 (treatment* OR procedure* OR surger* OR care)) OR ((dental OR t??th OR periodontal) NEAR/0 cleaning)) OR noft((((orthodontia OR orthodontolog* OR orthodonty) OR ((oral OR mouth) NEAR/1 surg*) OR ("community periodontal index of treatment needs" OR cpitn) OR ((mouth OR oral) NEAR/1 rehabilitation*) OR (((diagnos* OR examination*) NEAR/0 (oral OR dental))) OR (community NEAR/0 dentistry) OR (((dental OR periodontal OR t??th) NEAR/0 prophylaxis)) OR (((maxillo-mandibular OR maxillomandibular OR oral OR maxillofacial OR orthognathic OR jaw) NEAR/0 surg*) OR ((maxillo-mandibular OR maxillomandibular OR oral OR maxillofacial OR orthognathic) NEAR/1 procedure*)) OR ((oral NEAR/0 hygiene))))) OR noft(((("evidence-based" OR preventative OR cosmetic OR operative OR prosthetic OR reparative) NEAR/1 dentistry) OR (dental NEAR/0 (esthetic* OR aesthetic* OR floss OR scaling* OR brace* OR hygiene* OR polishing OR radiology OR bonding OR soldering* OR prosthetic*))) OR ((dental OR tooth) NEAR/2 filling*) OR ((dental OR tooth OR caries) NEAR/1 prevention) OR (dental NEAR/1 (implant* OR prosthes?s)) OR ((tooth OR teeth) NEAR/0 (whitening OR remineralization* OR polishing OR restoration* OR surger* OR preparation* OR removal*)) OR (dental NEAR/0 infection NEAR/0 control*) OR (tooth NEAR/1 (movement* OR care))) OR noft(fluoridation* OR (oral NEAR/0 medicine) OR (mouth NEAR/0 guard) OR (root NEAR/0 planing) OR ((oral OR maxillofacial) NEAR/2 pathology) OR (periodontal NEAR/0 medicine) OR (periodontal NEAR/0 (prosthesis OR prostheses)) OR (periodontal NEAR/0 prevention) OR (mouth NEAR/0 protection)))

**Key**

Su = subject heading

Noft = anywhere except full text

NEAR/x = terms within x words of each other (any order)

*= truncation

“ “ = phrase search

? = Mandatory wildcards (stands for exactly 1 character)

1. ISSG Search Filter Resource [Internet].  Glanville J, Lefebvre C, Wright K, editors.  York (UK):  The InterTASC Information Specialists' Sub-Group; 2008 [updated 2019 August 12; cited INSERT DATE].  Available from:  <https://sites.google.com/a/york.ac.uk/issg-search-filters-resource/home> [↑](#footnote-ref-1)
2. Search Filters for Various Databases [Internet]. The University of Texas Health Science Center at Houston (UTHealth), 2019. [cited 2019 12 13]. Available from: <https://libguides.sph.uth.tmc.edu/search_filters> [↑](#footnote-ref-2)
3. Observational Studies - EMBASE [Internet]. Ovid Expert Searches - Health Science. [cited 2019 12 16]. Available from: <http://resourcecenter.ovid.com/site/resources/expert_search/healthexp.html#OvidFilters> [↑](#footnote-ref-3)
4. Wilczynski NL, Marks S, Haynes RB. Search strategies for identifying qualitative studies in CINAHL. Qual Health Res., 2007; 17(5):705-10. [cited 2019 12 12]. Available from <https://journals.sagepub.com/doi/abs/10.1177/1049732306294515>. [↑](#footnote-ref-4)
5. Bramer, W. M, Giustini, D., de Jonge, G. B., Holland, L., Bekhuis, T. (2016). De-duplication of database search results for systematic reviews in EndNote. *Journal of the Medical Library Association, 104*(3), pp. 240-243. doi: [10.3163/1536-5050.104.3.014](https://dx.doi.org/10.3163%2F1536-5050.104.3.014). [↑](#footnote-ref-5)
