# Supplementary material for: The impact of dental care programs on healthcare system and societal outcomes: a scoping review
Source: BMC Health Serv Res. 2022 Dec 23;22:1574. doi: 10.1186/s12913-022-08951-x (PMC9780625; doi:10.1186/s12913-022-08951-x)
Supplement: Supplementary file 2 — Additional file 2. Quality assessment tool. [file 12913_2022_8951_MOESM2_ESM.docx]

**Additional file 2 – Quality assessment tool**

| **Quality Assessment Table** | | |
| --- | --- | --- |
| What type of study was this? | - Qualitative (move to qualitative questions) - Quantitative (move to quantitative questions) - Mixed-Method (move to mixed-method questions) | **Qualitative:** research that gathers information that is not numerical (e.g. interviews, focus groups etc.)  **Quantitative:** research that gathers numerical data that can be put into categories, or in rank order, or measured in units of measurement  **Mixed-method:** research that involves the mixing of quantitative and qualitative methods (e.g. exploratory design) |
| **Qualitative Quality Assessment** | | |
| Was there a clear statement of the research purpose/aims? | - Yes [text] - No [text] - Can’t Tell [text] | Consider the following to make a judgement: clarity of focus, explicit purpose given, supported by prior research |
| Does the collected data address the research question (or objectives)? | - Yes [text] - No [text] - Can’t Tell [text] | Consider whether the research and data collection strategy clearly described and was appropriate to address the research or objectives |
| Are the sources of qualitative data relevant to address the research question (objective)? | - Yes [text] - No [text] - Can’t Tell [text] | *Was the recruitment strategy appropriate to the aims of the research?* Consider whether (a) the selection of the participants is clear and appropriate to collect relevant and rich data; and (b) reasons why certain potential participants were chosen, or chose not to participate |
| Is the process for analyzing qualitative data relevant to address the research question (objective)? | - Yes [text] - No [text] - Can’t Tell [text] | *Was the data collected in a way that addressed the research issue?* Consider whether (a) the method of data collection is clear (e.g. focus group, interview); (b) the form of the data is clear (e.g. tape recordings, video material); (c) changes are explained when methods are altered during the study; (d) the qualitative data analysis addresses the question; and (e) the researcher has discussed saturation of data |
| Is appropriate consideration given to how findings relate to the context, e.g., the setting in which the data were collected? | - Yes [text] - No [text] - Can’t Tell [text] | Consider whether the study context and how findings relate to the context and characteristics of the context are explained (how findings are influenced by or influence the context) |
| Is appropriate consideration given to how findings relate to researchers’ influence, e.g., through their interactions with participants? | - Yes [text] - No [text] - Can’t Tell [text] | *Has the relationship between researcher and participants been adequately considered?* Consider whether (a) researchers critically explain how findings relate to their perspective, role, and interactions with participants (how the research process is influenced); (b) researcher’s role is influential at all stages; and (c) researchers explain their reaction to critical events that occurred during the study |
| Was the data analysis sufficiently rigorous? | - Yes [text] - No [text] - Can’t Tell [text] | Consider whether (a) data provided sufficient depth, detail and richness? (e.g. illustrative quotes); (b) an in-depth description of the analysis process (c) context described and taken into account in interpretation/results; (d) approaches taken to ensure robustness (e.g. multiple analysts, triangulation, member checking/participant validation of results) |
| Have ethical issues been taken into consideration? | - Yes [text] - No [text] - Can’t Tell [text] | Consider whether (a) study was approved by ethics committee; (b) sufficient details provided on how the research was explained to participants and whether ethical standards were maintained; (c) documentation of how autonomy, consent, confidentiality, anonymity were managed; and (d) documentation of any ethical dilemmas and how they were resolved |
| Is there a clear statement of findings? | - Yes [text] - No [text] - Can’t Tell [text] | Consider whether (a) the findings are explicit; (b) adequate discussion of the evidence both for and against the researchers arguments; (c) researcher has discussed the credibility of their findings (e.g. triangulations, respondent validations); and (d) findings are discussed in relation to research question |
| Was there evidence of study relevance and transferability? | - Yes [text] - No [text] - Can’t Tell [text] | Consider whether (a) there is a discussion of contribution of study to existing/prior knowledge, practice, and/or policy; (b) areas for future research identified; (c) limitations/weaknesses of study are clearly outlined; and (d) there is a discussion of whether or how the findings can be transferred to other populations or consideration of other ways the research may be used |
| **Quantitative Quality Assessment** | | |
| Was the study a randomized control trial? | - Yes (moves to randomized control questions) - No (moves to observational study questions) | **Randomized Control Trial**: individuals are allocated at random to a control or intervention group  **Observational studies** are ones where researchers observe the effect of a risk factor, diagnostic test, treatment or other intervention without trying to change who is or isn't exposed to it. Cohort studies and case control studies are two types of observational studies. |
| *Randomized Control Trials* | | |
| Was there a clear statement of the research purpose/aims? | - Yes [text] - No [text] - Can’t Tell [text] | Consider the following to make a judgement: clarity of focus, explicit purpose given, supported by prior research |
| Does the collected data address the research question (or objectives)? | - Yes [text] - No [text] - Can’t Tell [text] | Consider whether the research and data collection strategy clearly described and was appropriate to address the research or objectives |
| Is there a clear description of the randomization (or appropriate sequence generation)? | - Yes [text] - No [text] - Can’t Tell [text] | Consider whether researchers describe how the randomization or allocation is generated. A simple statement such as “we randomly allocated” or “using a randomized design” does not suffice. |
| Is there a clear description of the allocation concealment (or blinding when applicable)? | - Yes [text] - No [text] - Can’t Tell [text] - Not applicable | Consider whether (a) researchers and participants were unaware of the assignment sequence up to the point of allocation; or (b) researchers and participants are unaware of the group a participant is allocated to during the course of the study |
| Were the groups similar at the start of the trial? | - Yes [text] - No [text] - Can’t Tell [text] | Consider other factors that might affect the outcome such as age, sex, social class etc. |
| Are there complete outcome data (80% or above)? | - Yes [text] - No [text] - Can’t Tell [text] | Almost all of the participants contributed to almost all measures |
| Is there low withdrawal/drop-out (below 20%)? | - Yes [text] - No [text] - Can’t Tell [text] | Almost all of the participants completed the study |
| Was there evidence of study relevance and transferability? | - Yes [text] - No [text] - Can’t Tell [text] | Consider whether (a) there is a discussion of contribution of study to existing/prior knowledge, practice, and/or policy; (b) areas for future research identified; (c) limitations/weaknesses of study are clearly outlined; and (d) there is a discussion of whether or how the findings can be transferred to other populations or consideration of other ways the research may be used |
| *Observational Studies* | | |
| Was there a clear statement of the research purpose/aims? | - Yes [text] - No [text] - Can’t Tell [text] | Consider the following to make a judgement: clarity of focus, explicit purpose given, supported by prior research |
| Does the collected data address the research question (or objectives)? | - Yes [text] - No [text] - Can’t Tell [text] | Consider whether the research and data collection strategy clearly described and was appropriate to address the research or objectives |
| Are participants (organizations) recruited in a way that minimizes selection bias?(Explain how the study size was arrived at) | - Yes [text] - No [text] - Can’t Tell [text] | **Cohort:** consider whether the exposed (or with intervention) and non-exposed (or without intervention) groups are recruited from the same population  **Case-control:** consider whether (a) same inclusion and exclusion criteria were applied to cases and controls; (b) whether recruitment was done independently of the intervention or exposure status; and (c) was there a sufficient number of cases/controls selected? |
| Are measurements appropriate (clear origin, or validity known, or standard instrument; and absence of contamination between groups when appropriate) regarding the exposure/intervention and outcomes? | - Yes [text] - No [text] - Can’t Tell [text] | Consider whether (a) the variable are clearly defined and accurately measured; (b) the measurements are justified and appropriate for answering the research question, and (c) the measurements reflect what they are supposed to measure |
| In the groups being compared, are the participants comparable, or do researchers take into account (control for) the differences between the groups? | - Yes [text] - No [text] - Can’t Tell [text] | Have the authors taken account of the potential confounding factors in the design and/or analysis? Consider whether (a) the most important factors are taken into account in the analysis; (b) a table lists key demographic information comparing both groups, and there are no obvious dissimilarities between groups that may account for any differences in outcomes, or dissimilarities are taken into account in the analysis |
| Are there complete outcome data (80% or above), and, when applicable, an acceptable response rate (60% or above), or an acceptable follow-up rate for cohort studies (depending on the duration of follow-up)? | - Yes [text] - No [text] - Can’t Tell [text] |  |
| Did the authors report all outcomes? | - Yes [text] - No [text] - Can’t Tell [text] | Consider (a) if there is no evidence that outcomes were selectively reported (e.g. all relevant outcomes in the methods section are reported in the results section); (b) what are the bottom line results?; and (c) are the results adjusted for confounding, and might confounding still explain the association? |
| If a questionnaire was used to measure outcomes, was it appropriately validated and reliably tested? | - Yes [text] - No [text] - Can’t Tell [text] - Not applicable [text] | Consider if the questionnaire/focus group was appropriately validated and tested |
| Was there evidence of study relevance and transferability? | - Yes [text] - No [text] - Can’t Tell [text] | Consider whether (a) there is a discussion of contribution of study to existing/prior knowledge, practice, and/or policy; (b) areas for future research identified; (c) limitations/weaknesses of study are clearly outlined; and (d) there is a discussion of whether or how the findings can be transferred to other populations or consideration of other ways the research may be used |
| Was the study free of other problems that could put it at a high risk of bias? | - Yes [text] - No [text] - Can’t Tell [text] | Consider if you have any additional concerns about the design and/or conduct and reporting of this study |
| **Mixed-Method Quality Assessment** | | |
| Was there a clear statement of the research purpose/aims? | - Yes [text] - No [text] - Can’t Tell [text] | Consider the following to make a judgement: clarity of focus, explicit purpose given, supported by prior research |
| Does the collected data address the research question (or objectives)? | - Yes [text] - No [text] - Can’t Tell [text] | Consider whether the research and data collection strategy clearly described and was appropriate to address the research or objectives |
| Is the mixed methods research design relevant to address the qualitative and quantitative research questions (or objectives)? | - Yes [text] - No [text] - Can’t Tell [text] | Consider if the rationale for integrating qualitative and quantitative methods to answer the research question is explained. |
| Is the integration of qualitative and quantitative data (or results) relevant to address the research question (objective)? | - Yes [text] - No [text] - Can’t Tell [text] | There is evidence that data gathered by both research methods was brought together to form a complete picture, and answer the research question; authours explain when integration occurred; they explain how integration occurred and who participated in this integration |
| Is appropriate consideration given to the limitations associated with this integration, e.g., the divergence of qualitative and quantitative data (or results)? | - Yes [text] - No [text] - Can’t Tell [text] |  |
| Was there evidence of study relevance and transferability? | - Yes [text] - No [text] - Can’t Tell [text] | Consider whether (a) there is a discussion of contribution of study to existing/prior knowledge, practice, and/or policy; (b) areas for future research identified; (c) limitations/weaknesses of study are clearly outlined; and (d) there is a discussion of whether or how the findings can be transferred to other populations or consideration of other ways the research may be used |
| **Final Section** | | |
| Describe any important details that you believe were not extracted. | [text] |  |
