# Supplementary material for: The impact of dental care programs on healthcare system and societal outcomes: a scoping review
Source: BMC Health Serv Res. 2022 Dec 23;22:1574. doi: 10.1186/s12913-022-08951-x (PMC9780625; doi:10.1186/s12913-022-08951-x)
Supplement: Supplementary file 3 — Additional file 3. Outcome effectiveness. [file 12913_2022_8951_MOESM3_ESM.docx]

**Additional file 3 – Outcome effectiveness**

| **Author(s), year** | **Healthcare outcomes** | | | **Societal outcomes** | |
| --- | --- | --- | --- | --- | --- |
|  | Dental or healthcare services utilization/ attendance | Expenditure/cost of care | Averted treatments | Homelessness | Employability |
| 1. Abdus et al., 2019 ^(36)^ | + | + |  |  |  |
| 1. Beil et al., 2012 ^(37)^ |  | + |  |  |  |
| 1. Bergström et al., 2016 ^(38)^ |  | + | + |  |  |
| 1. Bhayat et al., 2016 ^(39)^ | + |  |  |  |  |
| 1. DiMarco et al., 2010 ^(40)^ | + |  |  |  |  |
| 1. Elani et al., 2020 ^(41)^ | +/0 |  |  |  |  |
| 1. Elani et al., 2020 ^(42)^ | + |  |  |  |  |
| 1. Kaakko et al., 2002 ^(43)^ | +/0 | 0 |  |  |  |
| 1. Khouja et al., 2020 ^(44)^ | 0 |  |  |  |  |
| 1. Kidd et al., 2020 ^(45)^ | + |  |  |  |  |
| 1. Lyu et al., 2020 ^(46)^ | + |  | + |  |  |
| 1. Maserejian et al., 2008 ^(47)^ | 0 |  |  |  |  |
| 1. McQuade et al., 2011 ^(48)^ | + |  |  |  |  |
| 1. Metsch et al., 2015 ^(49)^ | +/0 |  |  |  |  |
| 1. Moeller et al., 2020 ^(50)^ |  | ~~-~~ |  |  |  |
| 1. Nihtilä et al., 2013 ^(51)^ | + |  |  |  |  |
| 1. Nowak et al., 2014 ^(52)^ |  | + | + |  |  |
| 1. Nunez et al., 2013 ^(53)^ |  |  |  | + | + |
| 1. Pourat et al., 2020 ^(54)^ | + |  | + |  |  |
| 1. Rozier et al., 2010 ^(55)^ | + |  |  |  |  |
| 1. Sanjeevan et al., 2019 ^(56)^ | + |  |  |  |  |
| 1. Sen et al., 2013 ^(57)^ |  | 0 | + |  |  |
| 1. Singhal et al., 2013 ^(11)^ |  |  |  |  | 0 |
| 1. Singhal et al., 2016 ^(58)^ |  |  |  |  | +/0 |
| 1. Suominen et al., 2000 ^(59)^ |  |  | + |  |  |

+ indicates a favorable outcome, - indicates an unfavorable outcome, and 0 indicates no change. A combination indicates mixed results/inconclusive.
